# Emotions Toward Sustainable Innovations: A Matter of Value Congruence

**DOI:** 10.3389/fpsyg.2021.661314

**Published:** 2021-07-27

**Authors:** Nadja Contzen, Goda Perlaviciute, Pantea Sadat-Razavi, Linda Steg

**Affiliations:** ^1^Department of Psychology, Faculty of Behavioural and Social Sciences, University of Groningen, Groningen, Netherlands; ^2^Department Environmental Social Sciences, Eawag: Swiss Federal Institute of Aquatic Science and Technology, Duebendorf, Switzerland

**Keywords:** sustainable innovations, emotional responses, values, value implications, acceptability

## Abstract

Public resistance to sustainable innovations is oftentimes accompanied by strong negative emotions. Therefore, it is essential to better understand the underlying factors of emotions toward sustainable innovations to facilitate their successful implementation. Based on the Value-Innovation-Congruence model of Emotional responses (VICE model), we argue that positive and negative emotions toward innovations reflect whether innovations are congruent or incongruent with (i.e., support or threaten) people's core values. We tested our reasoning in two experimental studies (*N* = 114 and *N* = 246), by asking participants to evaluate innovations whose characteristics were either congruent or incongruent with egoistic values (study 1) or with biospheric values (study 1 and study 2). In line with the VICE model, we found overall that the more an innovation was perceived to have characteristics congruent with these values, and biospheric values in particular, the stronger positive and the weaker negative emotions they experienced toward the innovation, especially the more strongly people endorsed these values. Emotions, in turn, were related with acceptability of innovations. Our findings highlight that emotions toward innovations can have a systematic basis in people's values that can be addressed to ensure responsible decision-making on sustainable innovations.

## Introduction

Sustainable innovations^1^ can play a pivotal role in addressing societal challenges: new technologies, such as bio-energy with carbon capture and storage (BECCS), could help fight climate change, and new products, such as insect-based foods, could help combat climate change and increase food security. However, sustainable innovations may elicit strong public emotions that may affect public acceptability of these innovations and inhibit their implementation. Indeed, stronger positive and weaker negative emotions toward solar radiation management (Merk and Pönitzsch, 2017) and hydrogen fuel stations (Huijts et al., 2014) were related to stronger public acceptability of these innovations. Similarly, negative emotions toward genetically modified food explained opposition to their introduction (Scott et al., 2016), while negative emotions, namely disgust, toward recycled drinking water were related with resistance to consume it (Rozin et al., 2015). Emotions seem thus highly relevant for a successful and widespread implementation of sustainable innovations. It is therefore essential to better understand which factors and processes elicit such emotions, as this provides important insights into how to develop and implement sustainable innovations that elicit positive rather than negative emotions and are socially acceptable. In this paper, we experimentally test potential causes of emotions toward innovations based on the novel Value-Innovation-Congruence model of Emotional responses (VICE model; c.f. Perlaviciute et al., 2018). Moreover, we examine the relation between emotions toward innovations and acceptability of these innovations.

**Figure 1:**
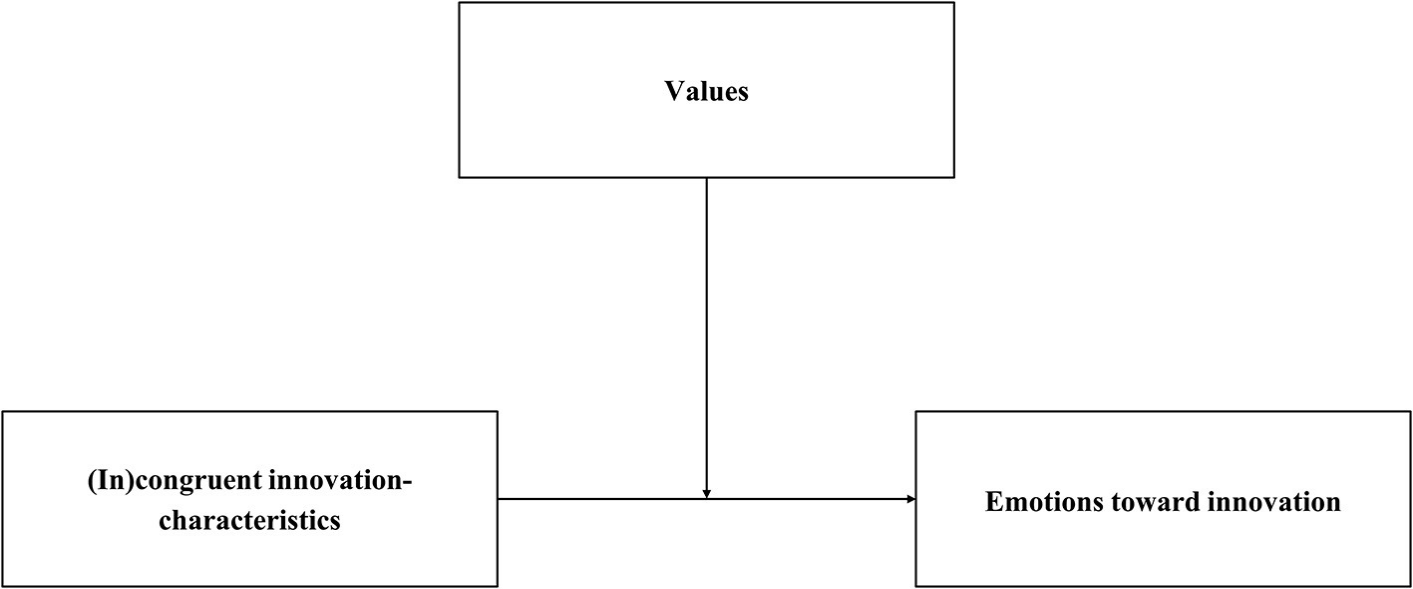
The Value-Innovation-Congruence model of Emotional responses (VICE model).

Integrating appraisal theories of emotions (Moors et al., 2013) and the theory of basic human values (Schwartz, 1992), our VICE model proposes that emotions toward sustainable innovations depend on the implications of innovations for people's values, notably their (in)congruence with people's values (see Figure 1; c.f. Perlaviciute et al., 2018; for related reasoning see Nelissen et al., 2007; Brosch and Sander, 2014). Values are general goals that people aspire in life that guide the selection and evaluation of behavior and events, across situations and time (Schwartz, 1992; Steg and de Groot, 2012; Dietz, 2015). Different characteristics of an innovation, such as its financial costs and benefits, health and safety risks, and consequences for nature and the environment, may be more or less congruent^2^ with different values people hold (e.g., Perlaviciute and Steg, 2015; c.f. Perlaviciute et al., 2018). The VICE model proposes that when an innovation's characteristics are congruent with, that is support, a person's values, positive emotions will result (see Figure 1; c.f. Perlaviciute et al., 2018). Conversely, when an innovation's characteristics are incongruent with, that is threaten, a person's values, negative emotions will emerge. This implies emotions toward innovations may reflect people's concerns that are based on their core values.

Sustainable innovations might be (in)congruent with four values in particular. Compared to conventional technologies and products, sustainable innovations are usually intended to have less negative and more positive implications for nature, such as lower CO_2_ emissions, as well as for others, such as improving public health. Such characteristics may be congruent with two types of values: biospheric values, reflecting valuing the protection of nature and the environment; and altruistic values, reflecting caring for the well-being of others (Steg et al., 2014b). At the same time, sustainable innovations are often more expensive, especially in the early stages of market launch, and may provide less comfort or involve some hassle, such as having to charge electric vehicles versus simply filling up conventional cars. Such characteristics may be *in*congruent with egoistic values, which reflect valuing personal resources such as wealth and status, and hedonic values, which reflect seeking pleasure and comfort (Steg et al., 2014b). While most people, across cultures and time, endorse each of the four values to a certain extent, people differ in how strongly they endorse them and which values they prioritize (Schwartz, 1992; Steg and de Groot, 2012; Dietz, 2015). Different people may therefore have different emotions toward one and the same innovation, depending on how strongly they endorse the values with which the innovation is either congruent or incongruent. If *sustainable* innovations have indeed characteristics congruent with biospheric and altruistic values and incongruent with egoistic and hedonic values, they may elicit stronger positive emotions the more strongly people endorse biospheric or altruistic values, and stronger negative emotions the more strongly people endorse egoistic or hedonic values. However, the emotions elicited by a *specific* sustainable innovation will depend on the innovation's *specific* characteristics and the extent to which they are (in)congruent with people's core values.

Importantly, while innovations have certain *given* characteristics, we argue that emotions depend on how people *perceive* these characteristics–which may not be in line with the given characteristics–and particularly the (in)congruence between the *perceived* characteristics and people's values. In fact, how people perceive an innovation's characteristics might depend on people's values as values have been found to influence cognitions, including beliefs, attitudes and preferences (e.g., Kalof et al., 1999; de Groot et al., 2013; Perlaviciute and Steg, 2015; c.f. Steg et al., 2014a). For example, a study on perceptions of nuclear energy revealed that the stronger people's egoistic values were, the more benefits, such as access to affordable energy, they perceived, while the stronger people's altruistic and biospheric values were, the more risks, such as environmental threats, they perceived (de Groot et al., 2013). It could be that values moderate the relation between given and perceived innovation-characteristics. Specifically, people may perceive characteristics of innovations more favorably when the given characteristics are congruent with their core values, and more unfavorably when they are incongruent with their core values.

**Figure 2:**
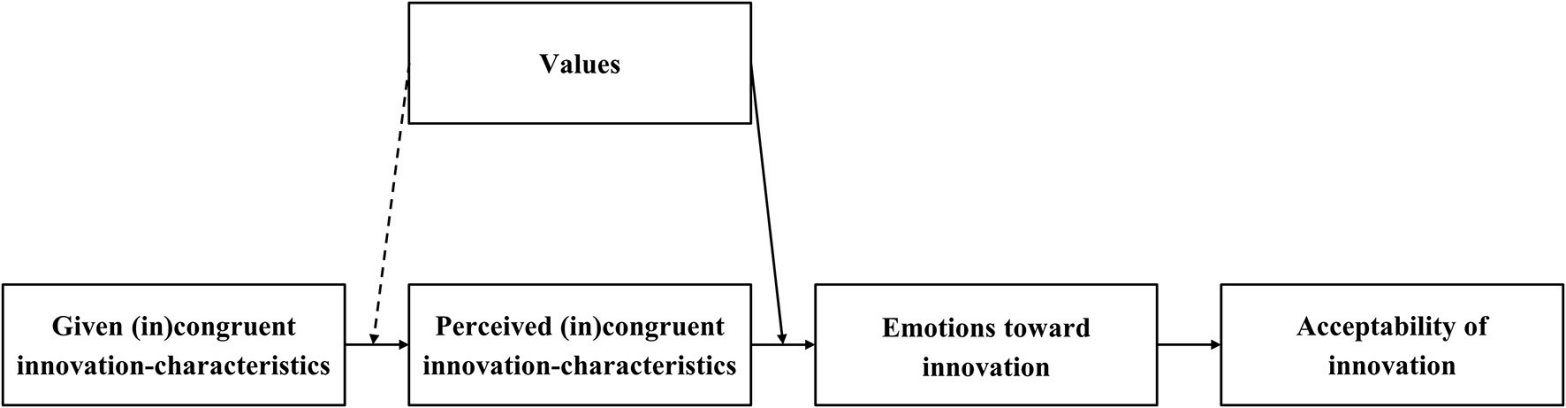
Working model of the present study, applying the VICE model with emotions explaining acceptability of (sustainable) innovations. The potential moderation effect of values on the effect of given on perceived innovation-characteristics (dashed arrow) will also be explored.

In this paper, we test for the first time the VICE model. Specifically, we test the effects of values and *perceived* innovation-characteristics on emotions toward (sustainable) innovations, as a function of the (in)congruence between values and perceived innovation-characteristics (see Figure 2). In line with the VICE model, we hypothesize that the more a person perceives an innovation as having characteristics congruent with specific values *and* the stronger the person endorses these specific values, the stronger positive emotions (H1a) and the weaker negative emotions (H1b) the person will experience toward the innovation.

We investigate these relations also for *given* innovation-characteristics^3^ instead of perceived innovation-characteristics and compare the respective results to assess whether an innovation's perceived characteristics are indeed essential to understand people's emotional responses toward innovations. Moreover, we test the effects of given innovation-characteristics on the corresponding perceived innovation-characteristics and explore whether these effects depend on people's values (see Figure 2).

Finally, we study whether emotions elicited by an innovation are related with acceptability of that innovation. We expect that the stronger the positive emotions (H2a) and the weaker the negative emotions (H2b) toward an innovation, the higher the acceptability of the innovation (see Figure 2).

We test the above hypotheses (and thus the VICE model) in two experimental studies. In study 1, we test the VICE model regarding emotional responses to innovative consumer products, while study 2 applies it to explain emotions toward a novel energy type. In the following, we present the methods and results of each study, followed by an overall discussion.

## Study 1: Testing the VICE Model to Explain Emotions Toward Innovative Consumer Products

### Materials and Methods

#### Procedure and Participants

We conducted an online study that comprised four experiments. Participants were recruited via the participant pool hosted by the Heymans Institute for Psychological Research at the University of Groningen. Participation was voluntary, following informed consent, and was rewarded with a token amount of €4. The study was conducted in strict compliance with the ethical principles of the American Psychological Association (American Psychological Association, 2017) and the World Medical Association Declaration of Helsinki (World Medical Association, 2018) and received ethical clearance by the Ethical Committee of Psychology at the University of Groningen.

**Figure 3:**
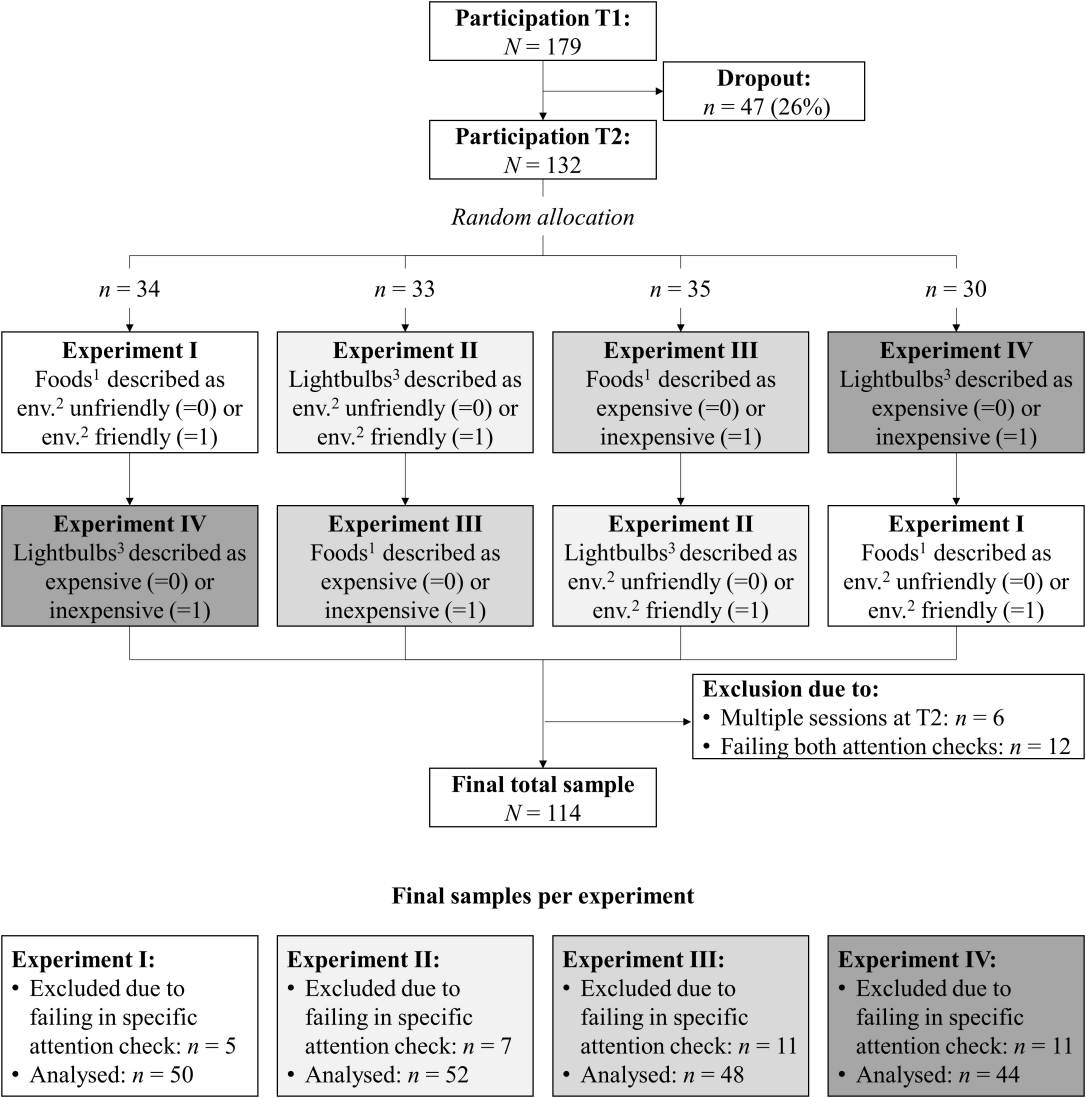
Participant flow and sample sizes in study 1. ^1^Microalgae-based foods. ^2^Environmentally. ^3^Nanophotonic lightbulbs.

Data were collected from April to June 2017 and consisted of two measurement points (see Figure 3 for the participant flow). At T1, after being introduced to the study topic and the procedure, the participants generated a unique identification code to match their responses at T1 and T2, completed a value measure, and reported their socio-demographic characteristics. Approximately 2 weeks later, at T2, participants were invited via email to participate in the main part of the study, which contained the four experiments and all remaining measures. Each participant randomly took part in two out of the four experiments (see Figure 3). In more detail, at T2, participants again received information on the procedure and generated the unique identification code. Next, they read information on one out of two innovative products containing the experimental manipulations, and answered questions regarding their emotions toward the respective innovation, its acceptability, and its perceived characteristics. These steps were then repeated for the second innovative product. Finally, attention checks were administered and participants were debriefed. Participation took around 5 min at T1 and around 15 min at T2. The entire study was in Dutch.

Of the 179 participants at T1, 132 (74%) participated also at T2. Contrary to the instructions, six participants completed the survey at T2 in more than one session, which could have affected the experimental manipulation; their responses were thus removed. Another 12 participants failed attention checks (see section Variables and Measures) of both experiments they took part in and were also removed, resulting in a final sample of 114 participants. They consisted of 92 women and 21 men (one missing). Their age ranged from 16 to 44 years (*M* = 22.34, *SD* = 3.53). Most respondents (89%) had followed higher education.

Of the final participants, some failed the attention check of only one of the two experiments they participated in and their responses were removed from the specific experiment; see Figure 3 for the final number of participants per experiment. As dropout from T1 to T2 was larger than expected and as we had to exclude more participants from analyses than anticipated, we achieved a slightly smaller sample than planned. We ran sensitivity power analysis with G^*^Power 3.1 in order to specify the effect size we were able to detect with the achieved sample size, given a power of 0.80 and an α = 0.05 (Erdfelder et al., 2005; Faul et al., 2009). As sample sizes differed per experiment, we ran the analysis per experiment. We report here the results for the most demanding analysis we applied, namely the hierarchical regression analysis testing H1a and H1b with four predictors in step 2 and six predictors in step 3 (see section Data Analysis Procedure). When testing our *directional* moderation hypotheses (i.e., one-tailed test of a *single* regression coefficient) in step 2 (i.e., testing four predictors), the smallest effect size we were able to detect ranged from *f*^2^ = 0.12 in experiment II to *f*^2^ = 0.14 in experiment IV. That is, the achieved sample sizes allowed us to detect medium effects but not small effects (Cohen, 1992). We also calculated the effect sizes we were able to detect in step 3, which explored the relations between values that were neither congruent nor incongruent with a product and emotions toward the product. The smallest effect size of a single regression coefficient we were able to detect in a two-tailed test with six predictors ranged from *f*^2^ = 0.16 in experiment II to *f*^2^ = 0.18 in experiment IV. That is, the achieved sample sizes allowed us to detect medium to large effects but not small effects.

#### Design and Manipulations

The experimental manipulation consisted of information on either microalgae-based foods (experiment I and III) or nanophotonic lightbulbs (experiment II and IV; see Table 1). These innovations were selected because people are mostly unfamiliar^4^ with them as they are either niche products (microalgae-based foods) or still in development (nanophotonic lightbulbs), which allowed us to experimentally manipulate the *given* innovation-characteristics as either incongruent or congruent with biospheric and egoistic values^5^, respectively.

**Table 1:** Overview of the four online experiments.

	**Innovation**	
**Manipulated innovation-characteristic**	**Microalgae-based foods**	**Nanophotonic lightbulbs**
Environmentally unfriendly (=0) vs. environmentally friendly (=1)	*Experiment I* (*n =* 50) Condition 1: Microalgae-based foods have, however, an important negative impact on nature: the cultivation and processing of microalgae emits much more CO_2_ than the cultivation and processing of meat and common meat replacers. If many people start to eat microalgae-based foods, climate change will be substantially increased. Condition 2: Microalgae-based foods have also an important positive impact on nature: the cultivation and processing of microalgae emits much less CO_2_ than the cultivation and processing of meat and common meat replacers. If many people start to eat microalgae-based foods, climate change will be substantially reduced.	*Experiment II* (*n =* 52) Condition 1: However, the new nanophotonic lightbulbs have an important negative impact on nature: the production of nanophotonic lightbulbs emits much more CO_2_ and uses much more hazardous substances than the production of fluorescent lamps and LED lamps. If fluorescent lamps and LED lamps are replaced in Europe by nanophotonic lightbulbs and if many people will use nanophotonic lightbulbs, climate change will substantially increase and more toxic waste will be released. Condition 2: What is more, the new nanophotonic lightbulbs have an important positive impact on nature: the production of nanophotonic lightbulbs emits much less CO_2_ and uses much less hazardous substances than the production of fluorescent lamps and LED lamps. If fluorescent lamps and LED lamps are replaced in Europe by nanophotonic lightbulbs and if many people will use nanophotonic lightbulbs, climate change will substantially decrease and less toxic waste will be released.
Expensive (=0) vs. inexpensive (=1)	*Experiment III* (*n* = 48) Condition 1: Microalgae-based foods are, however, financially very unattractive: the cultivation and processing of microalgae costs a lot; microalgae-based foods are thus much more expensive than meat and common meat replacers. By eating microalgae-based foods, households will lose a lot of money. Condition 2: Microalgae-based foods are also financially very attractive: the cultivation and processing of microalgae costs little; microalgae-based foods are thus much cheaper than meat and common meat replacers. By eating microalgae-based foods, households will save a lot of money.	*Experiment IV* (*n* = 44) Condition 1: However, the new nanophotonic lightbulbs are financially very unattractive: the production costs are very high, so that the lamps are four times more expensive than fluorescent lamps and LED lamps. If fluorescent lamps and LED lamps are replaced in Europe by nanophotonic lightbulbs and if households will use nanophotonic lightbulbs, they will lose a lot of money. Condition 2: What is more, the new nanophotonic lightbulbs are financially very attractive: the production costs are very low, so that the lamps are four times cheaper than fluorescent lamps and LED lamps. If fluorescent lamps and LED lamps are replaced in Europe by nanophotonic lightbulbs and if households will use nanophotonic lightbulbs, they will save a lot of money.

In experiments I (on microalgae-based foods) and II (on nanophotonic lightbulbs), we manipulated the innovations' environmental friendliness by describing them as either environmentally unfriendly or environmentally friendly, to render the innovations (in)congruent with biospheric values. In experiments III (on microalgae-based foods) and IV (on nanophotonic lightbulbs) we manipulated the innovations' (in)expensiveness by describing them as either expensive or inexpensive, to render the innovations (in)congruent with egoistic values. Each participant was randomly assigned to two out of the four experiments in such a way that they were presented with both innovations (microalgae-based foods and nanophotonic lightbulbs) once and with both types of manipulations [environmental friendliness and (in)expensiveness] once (see Figure 3). The order of the experiments was randomized per participant.

To make the innovation seem more credible and relevant to participants, all information texts started with some general information on the respective innovation, presenting it as having some important benefits and as being currently promoted (see Supplementary Material 1.1 for the full information texts). For microalgae-based foods, the information emphasized their major health benefits and stated a planned (but bogus) promotion campaign by the Dutch ministry of health aimed at increasing the consumption of microalgae-based foods. In case of nanophotonic lightbulbs, we highlighted their energy-efficient and comfortable use and mentioned a fictive effort by Europe's lighting industry association to introduce them on the European market with the aim to replace all existing lightbulbs on the market. The general information was followed by two sentences manipulating the congruence with biospheric or egoistic values (see Table 1 for the manipulation texts). The experiments were pretested (*N* = 45) and optimized accordingly.

#### Variables and Measures

Means and standard deviations of all study variables per experimental condition and their intercorrelations per experiment are presented in Supplementary Material 1.2.

*Values (T1)*. Participants' biospheric and egoistic values^6^ were measured with a short version of the Schwartz's value scale, which was developed and tested in previous research (Steg et al., 2014b). Participants were presented with a list of values accompanied by short descriptions and were asked to rate the importance of these values “as guiding principles in their lives” on a 9-point scale from −*1* = *opposed to my principles, 0* = *not important*, to *7* = *extremely important*. Each value was assessed with multiple items; the respective responses were averaged to form the respective value scale. Four items measured biospheric values, such as *Respecting the earth: harmony with other species* (Cronbach's α = 0.86). Egoistic values were measured with five items, such as *Social power: control over others, dominance* (Cronbach's α = 0.73).

*Perceived innovation-characteristics (T2)*. Participants evaluated the innovations' environmental friendliness and inexpensiveness on 9-point semantic differential scales ranging from *1* = *very harmful for the environment, 5* = *neither harmful nor good for the environment*, to *9* = *very good for the environment*, and *1* = *very expensive, 5* = *neither expensive nor cheap*, to *9* = *very cheap*.

*Emotions (T2)*. Participants reported how strongly they felt different positive and negative emotions when thinking about the innovations on a scale from *1* = *not at all* to *6* = *very strongly*. The emotions had been selected in a pilot study (see Supplementary Material 1.3 for more information). The positive emotions were comfortable, excited, happy, optimistic, relieved, and satisfied, and the negative emotions were afraid, angry, disappointed, disgusted, powerless, upset, and worried. For both innovations, exploratory factor analyses revealed that the positive and negative emotions loaded on separate factors and were averaged accordingly to form, per innovation, a positive emotions scale (Cronbach's α _Microalgae−based foods_ = 0.94; Cronbach's α _Nanophotonic lightbulbs_ = 0.90) and a negative emotions scale (Cronbach's α _Microalgae−based foods_ = 0.96; Cronbach's α _Nanophotonic lightbulbs_ = 0.90).

*Acceptability of innovation (T2)* was measured, per innovation, by means of eight 9-point semantic differential scales, ranging for example from *1* = *very negative, 5* = *neither negative nor positive*, to *9* = *very positive* (for all items see Supplementary Material 1.4). The items were averaged per innovation and formed consistent scales (Cronbach's α _Microalgae−based foods_ = 0.95; Cronbach's α _Nanophotonic lightbulbs_ = 0.97).

*Attention checks (T2)*. We administered two questions, one per innovation, to check whether participants had attentively read the manipulation texts (Tye-Williams, 2018). Per innovation, participants were presented with five statements and asked to select all statements that had appeared in the text about that innovation. For each experimental condition, there was only one correct statement. Participants passed the attention check if they selected the statement that was correct for the respective experimental condition, whether or not they had selected any additional, incorrect statements. Participants who failed in an attention check were excluded from all analyses for the respective experiment.

*Order of experiments*. To control for potential order effects, we added a dichotomous variable with −1 meaning that participants received the inexpensiveness manipulation first and the environmental friendliness manipulation second, and 1 meaning vice versa.

#### Data Analysis Procedure

All analyses were performed using IBM SPSS Statistics 27 with one exception. For all analyses that included a moderation test, we followed-up on significant interactions with simple slope tests using a template described by Dawson (2014). For the template, see www.jeremydawson.com/slopes.htm.

Following the working model of the present paper (see Figure 2), we first tested the effects of given, in the present study manipulated innovation-characteristics on the corresponding perceived innovation-characteristics, particularly to explore whether these effects depended on people's values. As in each experiment we manipulated specific innovation-characteristics as (in)congruent with specific values, we were particularly interested in whether these specific values affect perceptions. Therefore, in a step-wise approach, we first included values for which (in)congruence had been manipulated, and next the non-corresponding values for which (in)congruence had not been manipulated. In total, we ran four hierarchical multiple regression analyses, namely one per experiment. For all experiments, in step 1 the order of experiments was entered to control for potential order effects. For experiments I and II, we entered in step 2 the manipulated environmental friendliness, biospheric values and their interaction, and in step 3 egoistic values and their interaction with manipulated environmental friendliness. Similarly, for experiments III and IV, we entered in step 2 manipulated (in)expensiveness and egoistic values, and in step 3 biospheric values and their interaction with manipulated (in)expensiveness.

Next, we tested the VICE model, that is H1a and H1b. Again, as in each experiment we manipulated specific innovation-characteristics as (in)congruent with specific values, we were particularly interested in whether the corresponding perceived characteristics and values predict emotions. For experiments I and II, which included the manipulation of environmental friendliness, we tested whether perceived environmental friendliness together with biospheric (but not egoistic) values predict emotions, whereas for experiments III and IV, which included the manipulation of (in)expensiveness, we tested whether perceived (in)expensiveness together with egoistic (but not biospheric) values predict emotions.

In more detail, for experiments I and II in which we had manipulated innovations' environmental friendliness, we ran per experiment two hierarchical multiple regression analyses: one explaining positive and one explaining negative emotions. In step 1, we entered the order of experiments. Perceived environmental friendliness, biospheric values and their interaction were entered in step 2 to test H1a and H1b. Next, in step 3, we entered egoistic values and their interaction with perceived environmental friendliness. This allowed us to test whether indeed only the values (in)congruent with environmental friendliness (i.e., biospheric values) affected the emotions but not the other values (i.e., egoistic values), for which (in)congruence with environmental friendliness was not assumed.

For experiments III and IV in which we had manipulated innovations' (in)expensiveness, we followed a similar approach. Accordingly, we entered in step 2 perceived inexpensiveness, egoistic values, and their interaction, and in step 3 biospheric values and their interaction with perceived inexpensiveness.

The same analyses were run to investigate the effects of manipulated innovation-characteristics on emotions, moderated by values. Yet, manipulated environmental friendliness replaced perceived environmental friendliness, and manipulated inexpensiveness replaced perceived inexpensiveness.

For all of the above analyses, which contained the testing of a moderation effect, continuous predictors (i.e., personal values and perceived innovation-characteristics) were mean-centered prior to calculating the interaction terms and mean-centered scores were used in the respective analyses (Aiken et al., 1991). As samples differed between experiments (c.f. Table 1), the scores were mean-centered per experiment.

Finally, to test the relations between positive and negative emotions, and acceptability of innovations (H2a and H2b), we ran four hierarchical multiple regression analyses: one per experiment. In step 1, we entered the order of experiments, followed by positive and negative emotions in step 2.

In all analyses we assessed the significance of an estimate based on bias-corrected and accelerated (BCA) bootstrap confidence intervals^7^, using resamples of 5,000 (Wood, 2004). To test our *directional* moderation hypotheses (H1a and H1b), our *directional* relation hypotheses (H2a and H2b) and to test the *directional* effects of the experimental manipulations on the corresponding perceived innovation-characteristics, 90% confidence intervals, which correspond to a one-tailed significance test, were used to assess the respective estimates (Hayes and Preacher, 2014). For all other estimates, 95% confidence intervals were used, which correspond to a two-tailed significance test. Effect sizes were assessed in line with (Cohen, 1992).

### Results

#### Preliminary Analyses

Means and standard deviations of all study variables per experimental condition and their intercorrelations per experiment are presented in Supplementary Material 1.2. There were no significant differences between experimental conditions in value endorsement, suggesting the randomization to experimental conditions had been successful (see Tables SM1.2.A, SM1.2.B in Supplementary Material 1.2).

Indicating that our manipulations had been successful, participants in experiments I and II rated both innovations as significantly more environmentally friendly when they had been informed that the innovations are environmentally friendly than when they had received the opposite information (see Table SM1.2.A in Supplementary Material 1.2). Similarly, in experiments III and IV, both innovations were rated as more inexpensive when they had been described as inexpensive than when they had been described as expensive (see Table SM1.2.B in Supplementary Material 1.2). Interestingly, in none of the experiments was the perception of the other, non-corresponding innovation-characteristic affected by the manipulated innovation-characteristics (see Tables SM1.2.A, SM1.2.B in Supplementary Material 1.2). Specifically, manipulated environmental friendliness had no effect on perceived inexpensiveness, and manipulated inexpensiveness did not affect perceived environmental friendliness. Further, means and standard deviations of positive emotions were larger when the innovations had been presented as having positive characteristics than means and standard deviations of negative emotions when the innovations had been presented as having negative characteristics.

#### Effects of Manipulated Innovation-Characteristics on Corresponding Perceived Innovation-Characteristics and the Role of Values

We first tested the effects of given, in the present study manipulated innovation-characteristics on the corresponding perceived innovation-characteristics to explore whether these effects depend on people's values. In line with the preliminary analyses, in all four experiments the manipulated innovation-characteristics had large effects, in the expected directions, on the corresponding perceived characteristics (see Supplementary Material 1.5 for the results of the multiple regression analyses). In neither of the experiments did these effects depend on people's values, nor had values a direct effect on perceptions.

#### Testing the VICE Model: Perceived Innovation-Characteristics, Values, and Emotions Toward the Innovations

Tests of the VICE model are presented first regarding experiments I and II that manipulated innovations' environmental friendliness and next regarding experiments III and IV that manipulated innovations' (in)expensiveness.

##### Testing the VICE Model for Innovations Described as Environmentally (Un)friendly (Experiments I and II)

**Figure 4:**
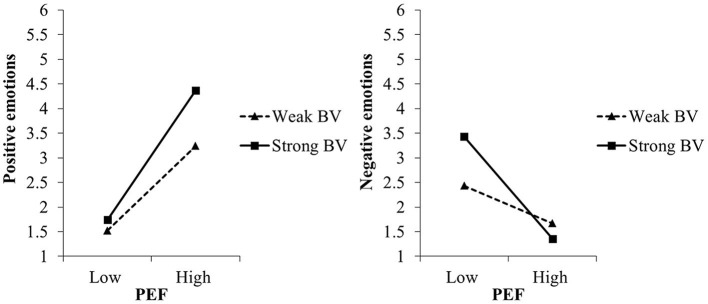
Moderating effects of biospheric values (BV) on the relation between perceived environmental friendliness (PEF) of and positive and negative emotions toward microalgae-based foods in experiment I, study 1. For the simple slope analyses, we used the estimates of step 2 of the regression analyses (c.f. Table 2).

In experiment I, in line with the VICE model, perceived environmental friendliness was significantly related with positive and negative emotions toward microalgae-based foods, dependent on biospheric values (see Table 2). More specifically, the more environmentally friendly people perceived microalgae-based foods to be, the stronger positive and the weaker negative emotions they reported. As expected, these relations were stronger, the stronger the biospheric values were, while they did not depend on egoistic values (for the simple slopes see Table 3 and Figure 4).

**Table 2:** Regression coefficients of innovations' perceived environmental friendliness, values, and their interactions on positive and negative emotions toward the innovations.

	**Experiment I (** ***n*** **= 50): Microalgae-based foods**										**Experiment II (** ***n*** **= 52): Nanophotonic lightbulbs**									
	**Positive emotions**					**Negative emotions**					**Positive emotions**					**Negative emotions**				
		**95% CI^a^^,^^b^**					**95% CI^a^^,^^b^**					**95% CI^a^^,^^b^**					**95% CI^a^^,^^b^**			
**IV^c^**	***B***	**LL**	**UL**	***SE***	**β**	***B***	**LL**	**UL**	***SE***	**β**	***B***	**LL**	**UL**	***SE***	**β**	***B***	**LL**	**UL**	***SE***	**β**
Step 2^d^																				
Constant	2.72	2.46	2.99	0.12		2.22	2.01	2.43	0.11		0.45	−0.12	1.05	0.32		1.87	1.68	2.06	0.10	
Order of exp.	0.20	−0.03	0.45	0.12	0.14	−0.07	−0.34	0.19	0.12	–0.07	−0.16	−0.42	0.11	0.14	–0.09	0.02	−0.17	0.20	0.09	0.02
PEF^e^	1.08	0.81	1.33	0.13	0.76	−0.71	−0.93	−0.47	0.11	–0.65	0.43	0.36	0.50	0.04	0.82	−0.75	−0.95	−0.56	0.10	–0.75
BV^f^	0.34	0.10	0.58	0.12	0.24	0.17	−0.06	0.43	0.11	0.16	0.06	−0.10	0.21	0.08	0.05	0.17	0.00	0.36	0.09	0.16
PEF^e^*BV^f^	0.22	0.02	0.42	0.12	0.16	−0.33	−0.51	−0.18	0.10	–0.31	0.19	0.02	0.35	0.10	0.11	−0.21	−0.35	−0.07	0.09	–0.20
Model fit	Δ*R*^2^ = 0.66, Δ*F*_(3, 45)_ *=* 29.51^***^ *R*^2^ = 0.66, *F*_(4, 45_) = 22.15^***^					Δ*R*^2^ = 0.53, Δ*F*_(3, 45)_ *=* 17.22^***^ *R*^2^ = 0.53, *F*_(4, 45)_ = 12.97^***^					Δ*R*^2^ = 0.70, Δ*F*_(3, 47)_ *=* 39.83^***^ *R*^2^ = 0.72, *F*_(4, 47)_ = 30.87^***^					Δ*R*^2^ = 0.58, Δ*F*_(3, 47)_ *=* 22.51^***^ *R*^2^ = 0.60, *F*_(4, 47_) = 17.36^***^				
Step 3																				
EV^g^	0.07	−0.17	0.30	0.12	0.05	0.33	0.07	0.58	0.12	0.30	0.07	−0.26	0.44	0.17	0.05	−0.10	−0.27	0.09	0.09	–0.10
PEF^e^*EV^g^	−0.07	−0.37	0.14	0.15	–0.05	−0.21	−0.43	0.11	0.12	–0.19	−0.10	−0.45	0.21	0.17	–0.06	0.02	−0.16	0.16	0.10	0.02
Model fit	Δ*R*^2^ = 0.00, Δ*F*_(2, 43)_ *=* 0.26 *R*^2^ = 0.67, *F*_(6, 43)_ = 14.36^***^					Δ*R*^2^ = 0.10, Δ*F*_(2, 43)_ *=* 6.21^**^ *R*^2^ = 0.64, *F*_(6, 43)_ = 12.72^***^					Δ*R*^2^ = 0.01, Δ*F*_(2, 45)_ *=* 0.55 *R*^2^ = 0.73, *F*_(6, 45)_ = 20.37^***^					Δ*R*^2^ = 0.01, Δ*F*_(2, 45)_ *=* 0.60 *R*^2^ = 0.61, *F*_(6, 45)_ = 11.58^***^				

a *BCA bootstrap confidence intervals based on 5,000 resamples*.

b *90% CIs are presented for the interaction between perceived environmental friendliness and biospheric values*.

c *Independent variable*.

d *Estimates of steps 1, in which we controlled for the influence of order of experiments on positive and negative emotions and for which the F-test of the overall significance of the model were non-significant, are not displayed here*.

e *Perceived environmental friendliness*.

f *Biospheric values*.

g *Egoistic values*.

** *p ≤ 0.01*,

*** *p ≤ 0.001*.

**Table 3:** Simple slopes of innovations' perceived environmental friendliness explaining positive and negative emotions toward the innovations at ±1 standard deviation (SD) of biospheric values.

	**Experiment I (** ***n*** **= 50): Microalgae-based foods**				**Experiment II (** ***n*** **= 52): Nanophotonic lightbulbs**			
	**Positive emotions**		**Negative emotions**		**Positive emotions**		**Negative emotions**	
	***B***	***t***	***B***	***t***	***B***	***t***	***B***	***t***
−1 *SD*	0.86	5.04^***^	−0.38	−2.42^*^	1.23	6.42^***^	−0.55	−4.08^***^
+1 *SD*	1.31	7.68^***^	−1.04	−6.57^***^	1.60	6.89^***^	−0.69	−7.16^***^

*For the simple slope analyses, we used the estimates of step 2 of the regression analyses (c.f. Table 2)*.

* *p ≤ 0.05*,

*** *p ≤ 0.001*.

When testing the effects of manipulated instead of perceived environmental friendliness on emotions, biospheric values moderated only the effects on negative but not on positive emotions. See Table SM1.6.A in Supplementary Material 1.6 for the results of the multiple regression analysis.

**Figure 5:**
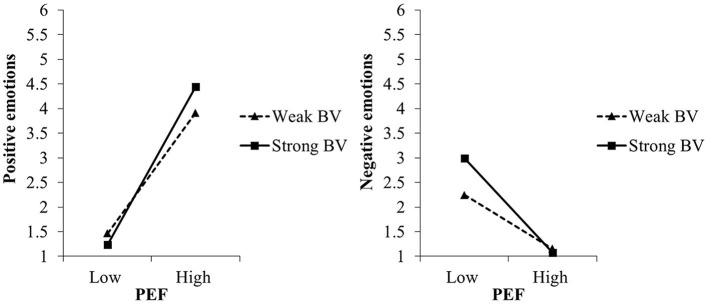
Moderating effect of biospheric values (BV) on the relation between perceived environmental friendliness (PEF) of and positive and negative emotions toward nanophotonic lightbulbs in experiment II, study 1. For the simple slope analyses, we used the estimates of step 2 of the regression analyses (c.f. Table 2).

Further supporting the VICE model, perceived environmental friendliness explained largely the emotions toward nanophotonic lightbulbs (experiment II), again moderated by biospheric but not egoistic values (Table 2). The more environmentally friendly people perceived nanophotonic lightbulbs to be, the stronger positive emotions and the weaker negative emotions they experienced, especially the stronger their biospheric values were (for the simple slopes see Table 3 and Figure 5). Noteworthy, biospheric values also moderated the effects of manipulated environmental friendliness on both positive and negative emotions (see Table SM1.6.A in Supplementary Material 1.6).

##### Testing the VICE Model for Innovations Described as (In)expensive (Experiments III and IV)

We subsequently tested the VICE model in experiments III and IV in which we had manipulated the innovations' (in)expensiveness; see Table 4 for the results. Of the regression models explaining positive emotions toward microalgae-based foods (experiment III), only step 3 had a significant overall fit. Yet against our expectation, perceived inexpensiveness was not significantly related to positive emotions^8^, even not among people with stronger egoistic values^9^. The relation depended neither on biospheric values, as expected. Unexpectedly, however, biospheric values were significantly and strongly related with positive emotions: the stronger the biospheric values were, the stronger positive emotions toward microalgae-based foods were reported, independent of perceived inexpensiveness (see Table 4). Results were similar when testing manipulated instead of perceived inexpensiveness (see Table SM1.6.B in Supplementary Material 1.6).

**Table 4:** Regression coefficients of innovations' perceived inexpensiveness, values, and their interactions on positive and negative emotions toward the innovations.

	**Experiment III (** ***n*** **= 44): Microalgae-based foods**										**Experiment IV (** ***n*** **= 48): Nanophotonic lightbulbs**									
	**Positive emotions**					**Negative emotions**					**Positive emotions**					**Negative emotions**				
		**95% CI^a^^,^^b^**					**95% CI^a^^,^^b^**					**95% CI^a^^,^^b^**					**95% CI^a^^,^^b^**			
**IV^c^**	***B***	**LL**	**UL**	***SE***	**β**	***B***	**LL**	**UL**	***SE***	**β**	***B***	**LL**	**UL**	***SE***	**β**	***B***	**LL**	**UL**	***SE***	**β**
Step 2^d^																				
Constant	2.88	2.52	3.31	0.19		1.52	1.36	1.71	0.10		3.36	3.06	3.62	0.16		1.38	1.26	1.52	0.07	
Order of exp.	0.00	−0.36	0.39	0.18	0.00	0.14	−0.01	0.31	0.09	0.24	0.05	−0.26	0.33	0.16	0.04	−0.02	−0.16	0.11	0.08	–0.04
PIE^e^	0.32	−0.04	0.73	0.18	0.26	−0.15	−0.34	0.05	0.09	–0.24	0.73	0.34	1.18	0.17	0.55	−0.21	−0.37	−0.06	0.07	–0.42
EV^f^	0.04	−0.36	0.38	0.19	0.03	0.09	−0.11	0.30	0.12	0.16	0.08	−0.23	0.40	0.15	0.06	0.10	−0.05	0.28	0.08	0.21
PIE^e^^*^EV^f^	0.02	−0.30	0.36	0.21	0.01	−0.10	−0.32	0.11	0.12	–0.17	0.39	0.11	0.81	0.19	0.29	−0.01	−0.12	0.16	0.09	–0.02
Model fit	Δ*R*^2^ = 0.07, Δ*F*_(3, 43)_ *=* 1.09 *R*^2^ = 0.07, *F*_(4, 43)_ = 0.82					Δ*R*^2^ = 0.11, Δ*F*_(3, 43)_ *=* 1.94 *R*^2^ = 0.16, *F*_(4, 43)_ = 2.08^t^					Δ*R*^2^ = 0.43, Δ*F*_(3, 39)_ *=* 9.78^***^ *R*^2^ = 0.43, *F*_(4, 39)_ = 7.34^***^					Δ*R*^2^ = 0.22, Δ*F*_(3, 39)_ *=* 3.69^*^ *R*^2^ = 0.22, *F*_(4, 39)_ = 2.77^*^				
Step 3																				
BV^g^	0.71	0.28	1.19	0.21	0.57	−0.13	−0.36	0.09	0.09	–0.22	0.10	−0.31	0.45	0.18	0.08	0.05	−0.16	0.22	0.10	0.10
PIE^e^^*^BV^g^	0.12	−0.30	0.71	0.22	0.09	0.08	−0.09	0.29	0.09	0.13	0.01	−0.48	0.30	0.25	0.00	−0.15	−0.40	0.10	0.12	–0.28
Model fit	Δ*R*^2^ = 0.27, Δ*F*_(2, 41)_ *=* 8.33^**^ *R*^2^ = 0.34, *F*_(6, 41)_ = 3.51^**^					Δ*R*^2^ = 0.07, Δ*F*_(2, 41)_ *=* 1.83 *R*^2^ = 0.23, *F*_(6, 41)_ = 2.05^t^					Δ*R*^2^ = 0.00, Δ*F*_(2, 37)_ *=* 0.15 *R*^2^ = 0.43, *F*_(6, 37)_ = 4.73^**^					Δ*R*^2^ = 0.08, Δ*F*_(2, 37)_ *=* 2.22 *R*^2^ = 0.30, *F*_(6, 37)_ = 2.70^*^				

a *BCA bootstrap confidence intervals based on 5,000 resamples*.

b *90% CIs are presented for the interaction between perceived inexpensiveness and egoistic values*.

c *Independent variable*.

d *Estimates of steps 1, in which we controlled for the influence of order of experiments on positive and negative emotions and for which the F-test of the overall significance of the model were non-significant, are not displayed here*.

e *Perceived inexpensiveness*.

f *Egoistic values*.

g *Biospheric values*.

*t = p ≤ 0.10*

* *p ≤ 0.05*,

** *p ≤ 0.01*,

*** *p ≤ 0.001*.

All regression models aimed at explaining negative emotions toward microalgae-based foods had only a marginally significant overall fit, indicating that neither perceived inexpensiveness nor its interaction with egoistic (or biospheric) values were meaningful predictors of negative emotions toward microalgae-based foods. The same is true for manipulated inexpensiveness (see Table SM1.6.B in Supplementary Material 1.6).

**Figure 6:**
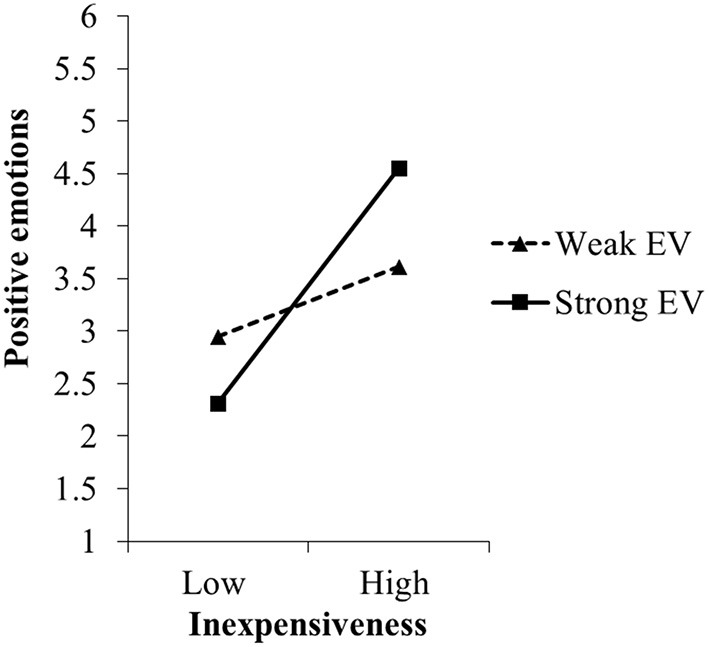
Moderating effect of egoistic values (EV) on the relation between perceived inexpensiveness of and positive emotions toward nanophotonic lightbulbs in experiment IV, study 1. For the simple slope analyses, we used the estimates of step 2 of the regression analyses (c.f. Table 4).

For nanophotonic lightbulbs (experiment IV), perceived inexpensiveness was strongly related to positive and negative emotions (Table 4): the more inexpensive people perceived nanophotonic lightbulbs to be, the stronger positive and the weaker negative emotions they reported. For positive emotions, the relation was moderated by egoistic but not biospheric values, thus supporting hypothesis H1a. Specifically, as expected, perceived inexpensiveness was only significantly related to positive emotions when egoistic values were strong (simple slope at +1 *SD*: *B* = 1.12, *t* = 5.23, *p* ≤ 0.001) but not when egoistic values were weak (simple slope at −1 *SD*: *B* = 0.33, *t* = 1.38, *p* = 0.176; see also Figure 6). When testing the effects of manipulated inexpensiveness on positive emotions, egoistic values did not moderate the effect (see Table SM1.6.B in Supplementary Material 1.6).

In contrast to our expectation (hypothesis H1b), egoistic values did not moderate the relation between perceived inexpensiveness and negative emotions. As expected, the relation did not depend on biospheric values either. The same results were obtained when testing manipulated instead of perceived inexpensiveness (see Table SM1.6.B in Supplementary Material 1.6).

#### Relations Between Emotions Toward the Innovations and Acceptability of the Innovations

In all four experiments, as expected, stronger positive (H2a) and weaker negative emotions (H2b) toward the innovations were related to higher acceptability of the innovations (see Table 5). Interestingly, the strength of relations differed somewhat between positive and negative emotions. In all four experiments, positive emotions were strongly related with acceptability, but negative emotions were only weakly to moderately related to acceptability. The amount of explained variance was somewhat higher in the two experiments in which environmental friendliness had been manipulated (experiments I and II), than in the two experiments in which (in)expensiveness had been manipulated (experiment III and IV).

**Table 5:** Regression coefficients of emotions toward innovations on acceptability of innovations.

	**Experiment I (** ***n*** **= 50) on environmental friendliness of microalgae-based foods**					**Experiment II (** ***n*** **= 52) on environmental friendliness of nanophotonic lightbulbs**					**Experiment III (** ***n*** **= 44) on inexpensiveness of microalgae-based foods**					**Experiment IV (** ***n*** **= 48) on inexpensiveness of nanophotonic lightbulbs**				
		**95% CI^a^^,^^b^**					**95% CI^a^^,^^b^**					**95% CI^a^^,^^b^**					**95% CI^a^^,^^b^**			
**IV^c^**	***B***	**LL**	**UL**	***SE***	**β**	***B***	**LL**	**UL**	***SE***	**β**	***B***	**LL**	**UL**	***SE***	**β**	***B***	**LL**	**UL**	***SE***	**β**
Step 2^d^																				
Constant	3.03	1.84	4.25	0.62		4.26	2.54	5.98	0.90		6.59	5.44	8.13	0.57		4.70	3.10	6.52	0.82	
Order of exp.	−0.32	−0.63	−0.03	0.16	–0.14	−0.01	−0.33	0.32	0.17	0.00	0.26	−0.05	0.56	0.14	0.19	0.10	−0.21	0.44	0.15	0.06
PE^e^	1.21	1.01	1.42	0.12	0.74	1.05	0.82	1.28	0.14	0.67	0.53	0.33	0.73	0.11	0.48	0.88	0.63	1.09	0.15	0.74
NE^f^	−0.53	−0.78	−0.31	0.15	–0.25	−0.89	−1.33	−0.47	0.27	–0.33	−1.01	−1.79	−0.68	0.35	–0.44	−0.58	−1.23	−0.02	0.37	–0.18
Model fit	Δ*R*^2^ = 0.80, Δ*F*_(2, 46)_ *=* 98.19^***^ *R^2^ =* 0.81*, F*_(3, 46)_ = 66.91***					Δ*R*^2^ = 0.79, Δ*F*_(2, 48)_ *=* 104.21^***^ *R^2^ =* 0.82*, F*_(3, 48)_ = 71.35^***^					Δ*R*^2^ = 0.51, Δ*F*_(2, 44)_ *=* 23.44^***^ *R^2^ =* 0.52, *F*_(3, 44)_ *=* 15.86^***^					Δ*R*^2^ = 0.62, Δ*F*_(2, 40)_ *=* 32.28^***^ *R^2^=*0.62*, F*_(3, 40)_ *=* 21.63^***^				

a *BCA bootstrap confidence intervals based on 5,000 resamples*.

b *90% CIs are presented for positive and negative emotions*.

c *Estimates of steps 1, which controlled for the influence of order of experiments on acceptability, and for which the F-test of the overall significance of the model were non-significant, are not displayed here*.

e *Positive emotions*.

f *Negative emotions*

*** *p ≤ 0.001*.

## Study 2: Testing the VICE Model to Explain Emotions Toward a Novel Energy Type

### Materials and Methods

#### Procedure and Participants

We conducted an online experiment among students of the University of Groningen. The majority of participants were recruited via an online platform for first year Bachelor psychology students and were rewarded with course credits (*n* = 203). The remaining participants were recruited in person at libraries and cafés of the University (*n* = 60). These participants could participate in a lottery to win a coffee voucher. Participation was voluntary, following informed consent. The study was conducted in compliance with the ethical principles of the American Psychological Association (American Psychological Association, 2017) and the World Medical Association Declaration of Helsinki (World Medical Association, 2018) and received ethical clearance by the Ethical Committee of Psychology at the University of Groningen.

Data were collected from April to May 2019. The online questionnaire was in English and started with information on the study topic and the procedure, asked for informed consent, and some sociodemographic information. The participants who had been recruited in person then completed a value measure. The other group of participants had previously completed the same measure as part of an earlier questionnaire administered among first year Bachelor psychology students. Next, all participants read information on an energy innovation containing the experimental manipulations, and answered questions regarding its perceived characteristics, their emotions toward the innovation and its acceptability. Finally, participants were debriefed. Participation took around 11 min.

Of the 263 people who participated in the study, 17 did not complete all variables of interest and were removed, resulting in a final sample of 246 participants. These were 161 women and 83 men (two missing). Their age ranged from 18 to 36 years (*M* = 20.92, *SD* = 2.30).

Again, we ran sensitivity power analysis^10^ with G^*^Power 3.1 (Erdfelder et al., 2005; Faul et al., 2009) for the most demanding analysis we had planned, namely the hierarchical regression analysis testing H1a and H1b with four predictors in step 2 and six predictors in step 3 (see section Data Analysis Procedure). When testing our *directional* moderation hypotheses (i.e., one-tailed test of a *single* regression coefficient) in step 2 (i.e., testing four predictors), the smallest effect size we were able to detect with a sample of *N* = 246, a power of 0.80 and an α = 0.05 was *f*^2^ = 0.02, which is a small effect (Cohen, 1992). In step 3, which explored the relations between values that were neither congruent nor incongruent with the studied innovation and emotions toward the innovation, the smallest effect size of a single regression coefficient we were able to detect in a two-tailed test with six predictors was *f*^2^ = 0.03 and thus also small.

#### Design and Manipulations

Study 2 also applied an experimental design in the form of information on an innovation. For study 2, we selected a novel energy type, namely algae-based biofuel, with which the participants were mostly unfamiliar^11^. This allowed us to experimentally manipulate the *given* innovation-characteristics as either incongruent or congruent with specific values. This time, we only manipulated the innovation's (in)congruence with biospheric values^12^. As in study 1, we manipulated the innovation's environmental friendliness by describing it as either environmentally unfriendly or environmentally friendly (see Table 6). The information texts of both conditions started again with some general information on the innovation. Specifically, in both conditions, algae was presented as a renewable energy source that is a potential solution to the world's growing energy demand and which emits less CO_2_ than fossil fuels (for the complete information texts provided to participants, see Supplementary Material 2.1). The information texts were pretested (*N* = 20) and optimized accordingly. Participants were randomly assigned to one of the experimental conditions.

**Table 6:** Texts containing the experimental manipulation.

**Experimental condition**	**Manipulation texts**
Environmentally unfriendly (=0)	However, it still **emits a substantial amount of CO**_**2**_ by the burning process that is required for energy generation. Additionally, in order to produce enough energy, algae requires huge areas of land and thus substantially contributes to the **clearing of forests** and **destruction of natural areas**. Another negative side effect is that algae **pollutes** water and land through poisonous fertilizers and pesticides that are used to grow it.
Environmentally friendly (=1)	Also, like any other plant it **reduces CO**_**2**_ from the atmosphere when grown in sunlight and releases oxygen, thereby depolluting the air. Additionally, algae can be grown in any type of water, including sweet water, natural water or wastewater, and therefore **does not require clearing of forests and destruction of natural areas**. Another positive side effect is that algae **depollutes the water** through absorbing and removing contaminants.

*Emphases are presented as in the original text. See Supplementary Material 2.1 for the complete information texts*.

#### Variables and Measures

Means and standard deviations of all study variables per experimental condition and their intercorrelations are presented in Supplementary Material 2.2.

*Values*. Participants' biospheric and egoistic values^13^ were measured as in study 1. Internal consistencies for both biospheric values (Cronbach's α = 0.86) and egoistic values (Cronbach's α = 0.73) were satisfactory to good.

*Perceived environmental friendliness*. Participants were asked how environmentally (un)friendly they thought algae-based biofuel was. The response scale ranged from *1* = *very harmful for the environment, 5* = *neither harmful nor good for the environment*, to *9* = *very good for the environment*.

*Emotions*. Participants reported how strongly they felt different emotions when thinking about the implementation of algae-based biofuel on a scale from *1* = *not at all* to *5* = *extremely*. We selected emotions in line with the 12-Point Affect Circumplex model of Core Affect (Yik et al., 2011). We included four positive *low* arousal emotions (i.e., satisfied, happy, comfortable, and at ease), three positive *high* arousal emotions (i.e., proud, euphoric, and excited), five negative *low* arousal emotions (i.e., sad, dissatisfied, unhappy, uncomfortable, and uneasy), and three negative *high* arousal emotions (i.e., nervous, fearful, and angry). Exploratory factor analysis revealed that the emotions formed two factors representing positive emotions (seven items) and negative emotions (eight items), while they did not separate into high and low arousal emotions. They were averaged accordingly to form a positive emotions scale (Cronbach's α = 0.93) and a negative emotions scale (Cronbach's α = 0.94).

*Acceptability of innovation* was measured with four 7-point semantic differential scales, ranging from 1 to 7, with the following poles: very negative to very positive; very bad to very good; very unnecessary to very necessary; and very unacceptable to very acceptable. The items were averaged and formed a consistent scale (Cronbach's α = 0.93).

*Type of recruitment and reward*. To control for potential effects of the type of participant recruitment and reward, we added a dichotomous variable with 1 meaning that participants were recruited online and received course credit in exchange for participation, and 2 meaning that they were recruited in person and could win a coffee voucher in exchange for participation.

#### Data Analysis Procedure

The analyses were performed analogous to those in study 1. As we had manipulated the innovation's (in)congruence with biospheric values, the testing of hypotheses H1a and H1b followed the analysis approaches used for experiments I and II in study 1. This time, we entered the type of recruitment and reward instead of the order of experiments as a control variable in step 1 of all hierarchical regression analyses.

### Results

#### Preliminary Analyses

Means and standard deviations of all study variables per experimental condition and their intercorrelations are presented in Supplementary Material 2.2. There were no significant differences between experimental conditions in value endorsement, suggesting the randomization to experimental conditions had been successful (see Table SM2.2.A in Supplementary Material 2.2).

Indicating that our manipulations had been successful, participants rated algae-based biofuel as significantly more environmentally friendly when they had been informed that the innovation is environmentally friendly than when they had received the opposite information (see Table SM2.2.A in Supplementary Material 2.2). Interestingly, participants reported stronger positive emotions toward algae-based biofuel when these had been presented as environmentally friendly compared to the reported strength of negative emotions when algae-based biofuel had been presented as environmentally unfriendly.

#### Effects of Manipulated Environmental Friendliness of Algae-Based Biofuel on Perceived Environmental Friendliness and the Role of Values

Again, we tested first the effect of the given innovation-characteristic, in the present study manipulated environmental friendliness, on the corresponding perceived innovation-characteristic to explore whether the effect depended on people's values. In line with the preliminary analyses, manipulated environmental friendliness had a large effect, in the expected direction, on perceived environmental friendliness (see Supplementary Material 2.3 for the results of the multiple regression analysis). As in study 1, this effect did not depend on people's values, nor had values a direct effect on perceived environmental friendliness.

#### Testing the VICE Model: Perceived Environmental Friendliness, Values, and Emotions Toward Algae-Based Biofuel

**Figure 7:**
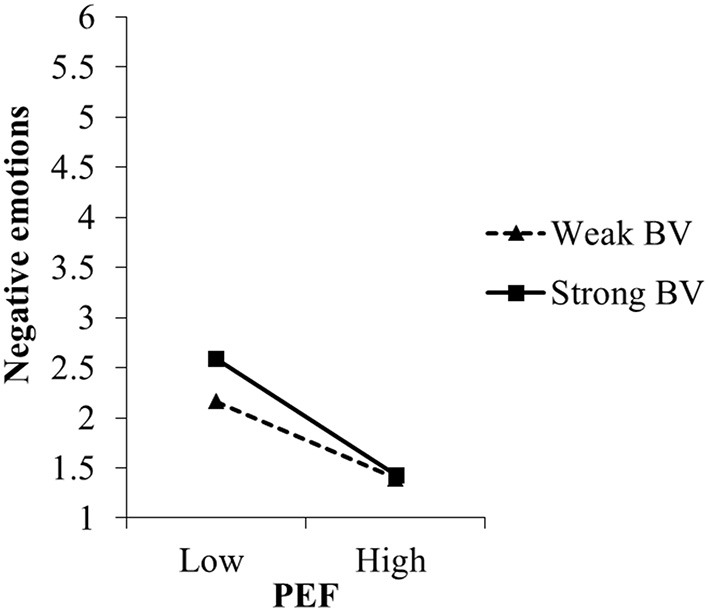
Moderating effect of biospheric values (BV) on the relation between perceived environmental friendliness (PEF) of and negative emotions toward algae-based biofuel in study 2. For the simple slope analyses, we used the estimates of step 2 of the regression analyses (c.f. Table 7).

Perceived environmental friendliness was strongly related with positive and negative emotions toward algae-based biofuel (see Table 7): the more environmentally friendly people perceived algae-based biofuel to be, the stronger positive and the weaker negative emotions they reported. For negative emotions, the relation was moderated by biospheric but not by egoistic values, thus supporting hypothesis H1b. Specifically, perceived environmental *un*friendliness was more strongly related with negative emotions when biospheric values were strong (simple slope at +1 *SD*: *B* = −0.58, *t* = −9.19, *p* ≤ 0.001) than when biospheric values were weak (simple slope at −1 *SD*: *B* = −0.38, *t* = 6.09, *p* ≤ 0.001; see also Figure 7). Noteworthy, biospheric values also moderated the effects of manipulated environmental friendliness on negative emotions (see Supplementary Material 2.4).

**Table 7:** Regression coefficients of perceived environmental friendliness of algae-based biofuel, values, and their interactions on positive and negative emotions toward algae-based biofuel.

	**Positive emotions**					**Negative emotions**				
		**95% CI^a^^,^^b^**					**95% CI^a^^,^^b^**			
**Independent variable**	***B***	**LL**	**UL**	***SE***	**β**	***B***	**LL**	**UL**	***SE***	**β**
Step 2^c^										
Constant	2.53	2.23	2.83	0.16		1.90	1.61	2.19	0.14	
Type of recruitment	0.11	−0.11	0.33	0.12	0.04	−0.12	−0.33	0.09	0.11	−0.06
PEF^d^	0.67	0.55	0.78	0.06	0.64	−0.48	−0.58	−0.38	0.05	−0.55
BV^e^	0.17	0.06	0.27	0.05	0.16	0.12	0.03	0.21	0.04	0.14
PEF^d^*BV^e^	0.05	−0.04	0.13	0.05	0.04	−0.10	−0.18	−0.02	0.05	−0.11
Model fit	Δ*R*^2^ = 0.43, Δ*F*_(3, 241)_ *=* 61.29^***^ *R*^2^ = 0.43, *F*_(4, 241)_ *=* 46.04^***^					Δ*R*^2^ = 0.35, Δ*F*_(3, 241)_ *=* 43.80^***^ *R*^2^ = 0.35, *F*_(4, 241)_ *=* 33.01^***^				
Step 3										
EV^f^	0.07	−0.03	0.16	0.05	0.07	0.02	−0.07	0.10	0.05	0.02
PEF^d^*EV^f^	−0.08	−0.19	0.01	0.05	–0.08	0.01	−0.09	0.13	0.05	0.01
Model fit	Δ*R*^2^ = 0.01, Δ*F*_(2, 239)_ *=* 2.29 *R*^2^ = 0.44, *F*_(6, 239)_ = 31.78^***^					Δ*R*^2^ = 0.00, Δ*F*_(2, 239)_ *=* 0.11 *R*^2^ = 0.35, *F*_(6, 239)_ = 21.88^***^				

a *BCA bootstrap confidence intervals based on 5,000 resamples*.

b *90% CIs are presented for the interaction between perceived environmental friendliness and biospheric values*.

c *Estimates of steps 1, which controlled for the influence of type of recruitment and reward on acceptability, and for which the F-test of the overall significance of the model were non-significant, are not displayed here*.

d *Perceived environmental friendliness*.

e *Biospheric values*.

f *Egoistic values*.

*** *p ≤ 0.001*.

In contrast to our expectation (hypothesis H1a), biospheric values did not moderate the relation between perceived environmental friendliness and positive emotions. Instead, biospheric values were significantly related to positive emotions: the stronger people's biospheric values were, the stronger positive emotions they reported. As expected, the relation between perceived environmental friendliness and positive emotions did not depend on egoistic values either. Noteworthy, when tested together with manipulated environment friendliness, biospheric values were not related with positive emotions, nor did they moderate the effect of manipulated environmental friendliness on positive emotions (see Supplementary Material 2.4).

#### Relations Between Emotions Toward and Acceptability of Algae-Based Biofuel

As expected, stronger positive (H2a) and weaker negative emotions (H2b) toward algae-based biofuel were related to higher acceptability of the innovations (see Table 8). As in study 1, positive emotions were more strongly related with acceptability than negative emotions.

**Table 8:** Regression coefficients of emotions toward algae-based biofuel on acceptability of algae-based biofuel.

		**95% CI of ***B***^a^^,^^b^**							
**Independent variable**	***B***	**LL**	**UL**	***SE***	**β**	**Δ*R*^**2**^**	**Δ*F***	***R^**2**^***	***F***
*Step 2* ^c^						0.72	318.97^***^	0.72	212.83^***^
Constant	4.12	3.59	4.66	0.28					
Type of recruitment	−0.03	−0.24	0.17	0.10	−0.01				
Positive emotions	0.80	0.71	0.88	0.05	0.60				
Negative emotions	−0.68	−0.78	−0.58	0.06	−0.43				

*N = 246*.

a *BCA bootstrap confidence intervals based on 5,000 resamples*.

b *90% CIs are presented for positive and negative emotions*.

c *Estimates of step 1, which controlled for the influence of type of recruitment and reward on acceptability, and for which the F-test of the overall significance of the model was non-significant, are not displayed here*.

*** *p ≤ 0.001*.

## Discussion

Sustainable innovations are more likely to be successfully implemented if they are accepted by the public. Public resistance to sustainable innovations is oftentimes accompanied by strong negative emotions. Therefore, it is essential to better understand the underlying factors and processes of emotions toward sustainable innovations. In this paper, we proposed, based on our VICE model, that values, together with an innovation's perceived characteristics, affect the emotions toward an innovation, depending on the congruence between values and innovation-characteristics (c.f. Perlaviciute et al., 2018). We tested the VICE model in two online studies containing five experiments in which we systematically varied innovation-characteristics that are (in)congruent with biospheric and egoistic values, respectively.

In both studies, we found support for the model's core assumption that people's values are at the basis of emotions toward sustainable innovations, especially, that the (in)congruence between the perceived characteristics of an innovation and people's biospheric values elicits emotions. Specifically, in study 1, the stronger people's biospheric values and the more they perceived the innovation as environmentally friendly, the stronger positive and the weaker negative emotions they experienced. These relations were found for both innovations, namely microalgae-based foods and nanophotonic lightbulbs. In study 2, these findings were mostly replicated. Specifically, study 2 showed that the stronger people's biospheric values and the more environmentally friendly people thought algae-based biofuel was, the weaker *negative* emotions were elicited. While perceived environmental friendliness of algae-based biofuel was also related with *positive* emotions, this relation was–different from our expectations–not moderated by biospheric values. Instead, we found a main effect for biospheric values: stronger biospheric values were related with stronger positive emotions toward algae-based biofuel, independent of the innovation's perceived environmental friendliness. A possible explanation for this finding is that the general information on algae-based biofuel we used in study 2 and which was provided in both conditions introduced algae-based biofuel as a renewable energy source that emits less CO_2_ than fossil fuels, which is congruent with biospheric values. As this general information rendered algae-based biofuels congruent with biospheric values, it may have elicited—independent of the additional information on the environmental (un)friendliness of algae-based biofuel that participants received in the respective experimental condition—stronger positive emotions toward algae-based biofuel particularly for those with stronger biospheric values.

Overall, our findings provide initial support for the VICE model and indicate that emotions toward sustainable innovations are not irrational or random (c.f. Cass and Walker, 2009) but have a systematic basis that reflects people's genuine concerns, namely an innovation's (in)congruence with people's important values. Further, our findings are in line with appraisal theories of emotions that assume that emotions toward a situation or object depend on whether the situation or object is perceived as congruent or incongruent with people's goals or concerns (e.g., Smith and Lazarus, 1993; Moors et al., 2013).

Further supporting the VICE model, in study 1, the stronger people's egoistic values and the more inexpensive people thought nanophotonic lightbulbs were, the more *positive* emotions were elicited. However, while perceived inexpensiveness of nanophotonic lightbulbs was also related with weaker *negative* emotions, this relation was not moderated by egoistic values. For microalgae-based foods, not only were relations between perceived inexpensiveness of microalgae-based foods and emotions independent of egoistic values, but perceived inexpensiveness of microalgae-based foods was not a meaningful predictor of *negative* emotions, and *positive* emotions were better explained by biospheric values than by perceived inexpensiveness of microalgae-based foods. Noteworthy, appraisal theories of emotions suggest that the perceived *relevance* of the appraised situation or object for a person's goals influences–in addition to goal-congruence–the person's emotional responses (e.g., Smith and Lazarus, 1993; Scherer, 2013), which could explain these findings. Innovation-characteristics (in)congruent with egoistic values, such as the researched (in)expensiveness of innovations, might particularly be personally relevant if people *must* use the innovation, for example if they actually have to buy and pay for it. The information on the two innovations we used in study 1 differed in this regard. The information on nanophotonic lightbulbs said that these lightbulbs might replace all existing lightbulbs on the European market wherefore participants may have thought they may be forced to buy them in future, thus adding personal relevance to the information on (in)expensiveness, especially for people with strong egoistic values. This may explain why egoistic values, together with perceived inexpensiveness, predicted at least positive emotions toward nanophotonic lightbulbs. The information on microalgae-based foods, on the other hand, only mentioned a planned promotion campaign, suggesting that its use is voluntary, thus reducing personal relevance from the information on (in)expensiveness. This might be the reason why perceived inexpensiveness predicted emotions toward microalgae-based foods only weakly and egoistic values had neither a moderating nor a direct effect on emotions. Future research could investigate to what extent the moderating effects of different values on the relation between innovation-characteristics and emotions toward innovations depend on the voluntariness of the innovation adoption.

Noteworthy, especially in study 1, values moderated the relation between perceived innovation-characteristics and emotions more consistently than the relation between manipulated innovation-characteristics and emotions. This indicates that to understand the basis of emotions toward innovations comprehensively, it is indeed important to consider the perceived characteristics of an innovation, which may differ from the given (here manipulated) innovation-characteristics.

In line with previous findings (e.g., Huijts et al., 2014; Rozin et al., 2015; Scott et al., 2016; Merk and Pönitzsch, 2017) stronger positive and weaker negative emotions toward innovations were in both studies related with higher acceptability of the innovations. Interestingly, the relations were weaker for negative than for positive emotions. This may be due to participants experiencing negative emotions only weakly, even in conditions describing innovations with negative characteristics, and this experience was quite consistent between participants (i.e., low variance in negative emotions), particularly in study 1. It is possible that negative emotions come primarily into play when an innovation's value incongruence and value relevance is large, which might be more strongly the case for more intrusive innovations, such as BECCS, than less intrusive innovations, such as the innovations investigated in the current studies. Future research could investigate, based on our model, emotions toward more intrusive innovations, maybe in comparison to emotions toward consumer products.

Our exploratory analyses revealed that the effect of given, in the present studies manipulated innovation-characteristics on corresponding perceived innovation-characteristics did not depend on people's values. This is in contrast to research showing that values affect people's perceptions systematically (e.g., Kalof et al., 1999; de Groot et al., 2013; Perlaviciute and Steg, 2015). While these studies focused on technologies and products that were already known to people, we introduced unknown innovations, for which participants had neither previous beliefs about their characteristics nor previous opinion about their acceptability. Probably due to this, our innovation descriptions affected perceptions exceptionally strongly, that is, participants evaluated the innovation-characteristics much in line with our descriptions. In contrast to research on, for example, conventional food products (e.g., chocolate; Schuldt et al., 2012) that found evaluative spill-over from manipulated product-characteristics (e.g., fair-trade) to other, non-corresponding product-characteristics (e.g., calorie content), in study 1, our manipulations only affected the perception of the corresponding innovation-characteristic but not the perception of the other, non-corresponding innovation-characteristic^14^. Our results might imply that values influence perceptions of innovation-characteristics mostly in the longer run, after people have established clear opinions, based on an innovation's characteristics and people's values. Specifically, particularly when opinions are established might perceptions of corresponding as well as non-corresponding characteristics be aligned to these value-based opinions (c.f. Perlaviciute and Steg, 2015). Future research could investigate in which stages of acceptability judgements values affect perceptions of innovations.

### Theoretical Implications, Limitations, and Perspectives for Future Research

To the best of our knowledge, our studies are the first that investigated systematically the value basis of emotions toward (sustainable) innovations and the first empirical test of the VICE model. By integrating appraisal theories of emotions (Moors et al., 2013) and the theory of human values (Schwartz, 1992), our VICE model allowed us to formulate specific hypotheses on people's emotions toward innovations, based on people's core values and the (in)congruence of innovations with these values (c.f. Perlaviciute et al., 2018; for related reasoning see also Nelissen et al., 2007; Brosch and Sander, 2014). Further, our findings on the relation between emotions toward and acceptability of innovations contribute to a broader research line that emphasizes the relevance of emotions and affect in pro-environmental decision-making more generally (e.g., Panno et al., 2015, 2020; Hahnel and Brosch, 2018; Brosch, 2021).

We tested our VICE model based on five experiments that considered innovations from three different product groups (i.e., foods, lightning products, and fuels) and their (in)congruence with two types of values (i.e., biospheric and egoistic values), which provides higher confidence in our findings. Focusing on innovations' (in)congruence with biospheric *and* egoistic values was especially relevant since sustainable innovations usually aim at benefiting the environment (i.e., congruence with biospheric values), but are oftentimes more expensive than conventional products and technologies (i.e., incongruence with egoistic values). Yet, sustainable innovations may also have characteristics that are (in)congruent with other values, such as altruistic and hedonic values. Such characteristics could be tested in future studies. Also, future research could consider other innovative products and extend to other types of innovations, including more intrusive innovations, such as carbon capture and storage or solar radiation management.

We found similar results in study 1 and study 2, which considered somewhat different types of positive and negative emotions toward the innovations. This provides further confidence in our findings as our results are not likely to depend on the specific emotions we included but apply more generally for negative and positive emotions that people may experience toward innovations.

The samples from both studies were comparably young and comprised mainly women. Future studies could replicate the findings among people with different demographic backgrounds, or with representative samples.

In study 1, testing our model in four experiments came at cost of rather small samples per experiment. This implied that we were able to detect medium effects but not small effects. The limitation is mitigated by the larger sample we achieved in study 2, which allowed us to detect also small effects. More importantly, since small effects have only limited practical relevance, even the achieved sample sizes in study 1 seem adequate to draw relevant practical conclusions on the importance of values, innovation-characteristics, and their interactions for emotions toward (sustainable) innovations.

Further, the data of study 1 could have been analyzed as a 2x2x2 factorial design. Instead, we analyzed the data separately per experiment due to the following reasons. First, the key variables, i.e., the perceived characteristics and values of interest, differed between the experiments manipulating innovations' environmental friendliness and the experiments manipulating innovations' (in)expensiveness. This implies that their measures differed also, which makes it inadequate to test them jointly. Second, the *general* information provided for the two innovations investigated in study 1, microalgae-based foods and nanophotonic lightbulbs, differed majorly from each other; as outlined above, this may have affected our results, especially in the experiments in which we had manipulated innovation's (in)congruence with egoistic values.

Also, the data used in study 2 was originally collected to answer questions beyond testing the VICE model (see Sadat-Razavi, 2019). Specifically, we aimed at testing whether an innovation's implementation status (i.e., whether an innovation is only considered for implementation or is already implemented), affects the relations specified in the VICE model. Therefore, we also manipulated the innovation's implementation status (see Supplementary Material 2.1). As the implementation status did not moderate the relations specified in the VICE model and as the factor is not relevant for the present purpose, we do not report on the findings in the present paper.

Next, while we assumed that specific innovation-characteristics are (in)congruent with specific values, we did not measure whether people perceived these value (in)congruencies. This could be considered in future studies.

The VICE model (Figure 1) and the study's working model (Figure 2) indicate causal relations, between perceived innovation-characteristics and emotions toward an innovation, and between emotions and acceptability of the innovation, respectively. However, we are, based on our data, not able to draw causal conclusions on these relations. Still, our research highlights the relevance of perceived characteristics, together with values, for the elicitation of emotions, and adds to the literature that emotions toward and acceptability of innovations are closely intertwined.

### Practical Implications

From a practical standpoint, our findings are highly relevant as we provide initial evidence that emotions toward sustainable innovations have a systematic basis that reflects people's genuine concerns, namely an innovation's (in)congruence with people's core values. Emotions and their underlying factors may thus provide important insights for the decision-making on sustainable innovations that could be considered from the earliest decision-stages on to improve the development and implementation of potentially promising sustainable innovations. This is in contrast to the assumption of many practitioners, including R&D professionals, politicians, and project managers, that emotions are irrational or random reactions and thus an illegitimate base of decision-making (c.f. Cass and Walker, 2009).

Importantly, our findings highlight the relevance of considering both positive and negative emotions in practice, not least as we found that particularly positive emotions were related with acceptability of innovations. Current practice, in contrast, is to consider mainly negative emotions, if at all, probably because of their obstructive potential (c.f. Perlaviciute et al., 2018). Balanced decision-making on the development and implementation of sustainable decision, however, should consider equally the positive and negative emotions toward innovations and their underlying factors.

Of further practical relevance, our research provides a framework to analyze the basis of emotions toward innovations to be considered in decision-making, that is, people's perceptions of innovation-characteristics and their core values. Analyzing emotions and their basis could unveil innovations' value incongruence, and might give opportunities to remove or at least reduce such incongruence. In this light, our model could be applied to investigate systematically the emotions toward innovations that are discussed controversially in society, such as carbon capture and storage or solar radiation management. Importantly, considering emotions and their basis could also unveil value congruence, which is equally relevant as it provides important information on the core reasons for implementing an innovation.

## Conclusion

In two experimental studies we investigated systematically the value basis of emotions toward (sustainable) innovations and found initial evidence that people's core values, together with the extent to which the perceived innovation-characteristics are (in)congruent to these values, affect emotions toward an innovation. These results indicate that emotions toward sustainable innovations have a systematic basis that reflects people's genuine concerns. This implies that emotions and their underlying factors may provide important insights for the decision-making on sustainable innovations.

## Data Availability Statement

The datasets generated for this study are available on request to the corresponding author.

## Ethics Statement

The studies involving human participants were reviewed and approved by the Ethical Committee of Psychology of the University of Groningen. The participants provided their written informed consent to participate in this study.

## Author Contributions

NC, GP, and LS contributed to conception and design of study 1, while study 2 was conceived and designed by PS-R and GP, based on study 1. NC organized the data collection of study 1, performed the statistical analysis of both studies, and wrote the first draft of the manuscript. PS-R organized the data collection of study 2. All authors discussed and interpreted the results, contributed to manuscript revision, and read and approved the submitted version.

## Conflict of Interest

The authors declare that the research was conducted in the absence of any commercial or financial relationships that could be construed as a potential conflict of interest.

## Publisher's Note

All claims expressed in this article are solely those of the authors and do not necessarily represent those of their affiliated organizations, or those of the publisher, the editors and the reviewers. Any product that may be evaluated in this article, or claim that may be made by its manufacturer, is not guaranteed or endorsed by the publisher.

## Footnotes

^1^The author(s) do not take any stances on whether the innovations mentioned in this article are sustainable and whether they should be implemented.

^2^The congruence or incongruence may not only be related to the innovation (i.e., the technology or product) itself, but may also be related to, among others, the decision-making process and the context in which the innovation is proposed or implemented (e.g., the region or landscape in which a technology is sited).

^3^In the present study, given innovation-characteristics are represented by experimentally manipulated innovation-characteristics.

^4^When asked about the familiarity of the innovations, participants rated them as rather unfamiliar (microalgae-based foods: *M* = 3.19, *SD* = 1.74; nanophotonic lightbulbs: *M* = 3.71, *SD* = 1.95) on a scale from *1* = *very unfamiliar* to *9* = *very familiar*.

^5^(In)congruence with altruistic and hedonic values were outside the scope of the present study.

^6^Altruistic and hedonic values, though measured as part of the values scale, are not further described here, because the experimental manipulations did not target these values.

^7^Compared with conventional significance tests, bootstrap confidence intervals quantify the uncertainty as well as the accuracy of estimates and bootstrapping is a more robust approach (Wood, 2004).

^8^The estimates for perceived inexpensiveness in step 3 were: *B* (95% CI) = 0.25 (−0.04, 0.73); β = 0.20.

^9^The estimates for the interaction between perceived inexpensiveness and egoistic values in step 3 were: *B* (90% CI) = 0.23 (−0.07, 0.53); β = 0.18.

^10^The data was originally collected to answer questions beyond testing the VICE model (see Sadat-Razavi, 2019). Specifically, the original study aimed at testing whether an innovation's implementation status (i.e., whether an innovation is only considered for implementation or is already implemented) affects the relations specified in the VICE model. Therefore, the a priori power calculation we had originally conducted is not informative for the present purpose.

^11^When asked about the familiarity of algae-based biofuel, participants rated it as very unfamiliar (*M* = 1.54, *SD* = 1.45) on a scale from *0* = *not familiar at all* to *7* = *very familiar*.

^12^Extending the experiments applied in study 1, we additionally manipulated the innovation's implementation status (see Sadat-Razavi, 2019). That is, we described the innovation either as being only considered for implementation or as already being implemented (see Supplementary Material 2.1). The study thus followed a 2x2 between-subjects design. As the additional factor is not relevant for the present purpose and as it did not moderate the relations of interest, we do not report it in the present paper.

^13^Altruistic and hedonic values, though measured as part of the values scale, are not further described here, because they were not used in any of the analyses.

^14^In study 2, we did not measure any non-corresponding innovation-characteristic.
