# Supplementary material for: Emotions Toward Sustainable Innovations: A Matter of Value Congruence
Source: Front Psychol. 2021 Jul 27;12:661314. doi: 10.3389/fpsyg.2021.661314 (PMC8354012; doi:10.3389/fpsyg.2021.661314)
Supplement: Supplementary file 1 [file Data_Sheet_1.pdf]

Supplementary Material

1 Supplementary Material of Study 1

1.1 Information Texts

Table SM1.1.A. Information provided on microalgae-based foods in experiments I and III.

Section/Experiment	Condition	Text
General introduction	All conditions	To promote public health, earlier this year the Netherlands Nutrition Centre recommended to eat less meat. The Nutrition Centre advises to eat meat maximally twice a week and to eat other protein sources for the remaining meals. Therefore, the Ministry of Public Health, Welfare and Sport will soon launch a nationwide campaign to promote the consumption of a new alternative protein source: microalgae. Microalgae are much better for health than meat or common meat replacers, such as soybeans: they contain not only much proteins but also more vital nutrients, such as minerals and vitamins A, B, C and E, which our body needs to work properly.
Experiment I	Condition 1: Environmental-unfriendliness	Microalgae-based foods have, however, an important negative impact on nature: the cultivation and processing of microalgae emits much more CO_2 than the cultivation and processing of meat and common meat replacers. If many people start to eat microalgae-based foods, climate change will be substantially increased.
	Condition 2: Environmental friendliness	Microalgae-based foods have also an important positive impact on nature: the cultivation and processing of microalgae emits much less CO_2 than the cultivation and processing of meat and common meat replacers. If many people start to eat microalgae-based foods, climate change will be substantially reduced.
Experiment III	Condition 1: Expensiveness	Microalgae-based foods are, however, financially very unattractive: the cultivation and processing of microalgae costs a lot; microalgae-based foods are thus much more expensive than meat and common meat replacers. By eating microalgae-based foods, households will lose a lot of money.
	Condition 2: Inexpensiveness	Microalgae-based foods are also financially very attractive: the cultivation and processing of microalgae costs little; microalgae-based foods are thus much cheaper than meat and common meat replacers. By eating microalgae-based foods, households will save a lot of money.

Table SM1.1.B. Information provided on nanophotonic lightbulbs in experiments II and IV.

Section/experiment	Condition	Text
General introduction	All conditions	The European commission decided in 2008 to ban traditional lightbulbs because they consume a lot of energy. They have to be replaced by energy-efficient lamps, such as fluorescent lamps and LED lamps, which consume five to ten times less energy than traditional lightbulbs. Both lamps have, however, a disadvantage: they produce a whiter light than traditional lightbulbs, which emit a warm, yellow glow. At the moment, LightingEurope, an industry association of the European lighting industry, request approval of the introduction of a new type of lamp in Europe: nanophotonic lightbulbs. These lamps shall not only replace traditional lightbulbs but also fluorescent lamps and LED lamps. The new nanophotonic lightbulbs are exactly as energy-efficient as fluorescent lamps and LED lamps, but they emit a similarly warm, yellow light as traditional lightbulbs.
Experiment II	Condition 1: Environmental-unfriendliness	However, the new nanophotonic lightbulbs have an important negative impact on nature: the production of nanophotonic lightbulbs emits much more CO_2 and uses much more hazardous substances than the production of fluorescent lamps and LED lamps. If fluorescent lamps and LED lamps are replaced in Europe by nanophotonic lightbulbs and if many people will use nanophotonic lightbulbs, climate change will substantially increase and more toxic waste will be released.
	Condition 2: Environmental friendliness	What is more, the new nanophotonic lightbulbs have an important positive impact on nature: the production of nanophotonic lightbulbs emits much less CO_2 and uses much less hazardous substances than the production of fluorescent lamps and LED lamps. If fluorescent lamps and LED lamps are replaced in Europe by nanophotonic lightbulbs and if many people will use nanophotonic lightbulbs, climate change will substantially decrease and less toxic waste will be released.
Experiment IV	Condition 1: Expensiveness	However, the new nanophotonic lightbulbs are financially very unattractive: the production costs are very high, so that the lamps are four times more expensive than fluorescent lamps and LED lamps. If fluorescent lamps and LED lamps are replaced in Europe by nanophotonic lightbulbs and if households will use nanophotonic lightbulbs, they will lose a lot of money.
	Condition 2: Inexpensiveness	What is more, the new nanophotonic lightbulbs are financially very attractive: the production costs are very low, so that the lamps are four times cheaper than fluorescent lamps and LED lamps. If fluorescent lamps and LED lamps are replaced in Europe by nanophotonic lightbulbs and if households will use nanophotonic lightbulbs, they will save a lot of money.

1.2 Descriptive Statistics and Intercorrelations of Variables Tested in Study 1

Table SM1.2.A. Means and standard deviations of values, perceived innovation-characteristics, positive and negative emotions, and acceptability in experiments I and II, which manipulated environmental friendliness.

	Condition[1] (*n*)	*M*	*SD*	*t*
Experiment I on microalgae-based foods				
Biospheric values	1 (25)	3.82	1.64	*t*(48) = -0.25
	2 (25)	3.93	1.43	
Egoistic values	1 (25)	2.63	1.21	*t*(48) = 0.09
	2 (25)	2.60	1.29	
Perceived environmental friendliness	1 (25)	1.84	1.65	*t*(48) = -15.27***
	2 (25)	7.80	1.04	
Perceived inexpensiveness	1 (25)	3.96	1.67	*t*(48) = 0.33
	2 (25)	3.80	1.73	
Positive emotions	1 (25)	1.85	1.16	*t*(48) = -5.73***
	2 (25)	3.64	1.05	
Negative emotions	1 (25)	2.80	1.15	*t*(36.84) = 4.55***
	2 (25)	1.61	0.62	
Acceptability	1 (25)	3.54	1.79	*t*(48) = -6.79***
	2 (25)	6.75	1.54	
Experiment II on nanophotonic lightbulbs				
Biospheric values	1 (24)	3.95	1.39	*t*(50) = -0.95
	2 (28)	4.31	1.38	
Egoistic values	1 (24)	2.81	1.28	*t*(50) = 10.4
	2 (28)	2.48	0.99	
Perceived environmental friendliness	1 (24)	1.54	0.83	*t*(50) = -18.61***
	2 (28)	7.61	1.40	
Perceived inexpensiveness	1 (24)	4.42	1.61	*t*(50) = 1.16
	2 (28)	3.93	1.41	
Positive emotions	1 (24)	1.22	0.39	*t*(33.85) = -12.77***
	2 (28)	4.17	1.15	
Negative emotions	1 (24)	2.56	0.98	*t*(31.11) = 6.24***
	2 (28)	1.20	0.45	
Acceptability	1 (24)	2.93	1.16	*t*(50) = -4.93***
	2 (28)	7.86	1.13	

[1] Condition 1: Environmentally unfriendly. Condition 2: Environmentally friendly. * $p \leq .05$. ** $p \leq .01$. *** $p \leq .001$.

Table SM1.2.B. Means and standard deviations of values, perceived innovation-characteristics, positive and negative emotions, and acceptability in experiments III and IV, which manipulated inexpensiveness.

	Condition[1] (*n*)	*M*	*SD*	*t*
Experiment III on microalgae-based foods				
Egoistic values	1 (27)	2.33	1.12	$t(46) = -1.59$
	2 (21)	2.88	1.24	
Biospheric values	1 (27)	4.23	1.54	$t(46) = 0.79$
	2 (21)	3.89	1.37	
Perceived inexpensiveness	1 (27)	1.93	0.96	$t(28.99) = -11.08$***
	2 (21)	6.67	1.77	
Perceived environmental friendliness	1 (27)	6.74	1.72	$t(46) = -0.78$
	2 (21)	7.10	1.64	
Positive emotions	1 (27)	2.70	1.26	$t(46) = -1.13$
	2 (21)	3.11	1.21	
Negative emotions	1 (27)	1.62	0.72	$t(46) = 1.51$
	2 (21)	1.36	0.35	
Acceptability	1 (27)	6.48	1.37	$t(46) = -0.69$
	2 (21)	6.76	1.40	
Experiment IV on nanophotonic lightbulbs				
Egoistic values	1 (19)	2.82	1.15	$t(42) = 0.62$
	2 (25)	2.58	1.34	
Biospheric values	1 (19)	4.07	1.51	$t(42) = 1.59$
	2 (25)	3.34	1.48	
Perceived inexpensiveness	1 (19)	2.26	1.05	$t(42) = -9.25$***
	2 (25)	6.64	1.85	
Perceived environmental friendliness	1 (19)	6.16	1.98	$t(42) = -1.79$
	2 (25)	7.12	1.59	
Positive emotions	1 (19)	2.73	1.07	$t(42) = -3.07$***
	2 (25)	3.85	1.29	
Negative emotions	1 (19)	1.65	0.60	$t(23.74) = 3.22$**
	2 (25)	1.18	0.27	
Acceptability	1 (19)	6.04	1.77	$t(27.34) = -3.13$**
	2 (25)	7.47	1.04	

[1] Condition 1: Expensive. Condition 2: Inexpensive. * $p \leq .05$. ** $p \leq .01$. *** $p \leq .001$.

Table SM1.2.C. Correlations between manipulated environmental friendliness, values, perceived environmental friendliness, positive and negative emotions, and acceptability in experiment I (above diagonal) and experiment II (below diagonal)

	Manipulated environmental friendliness (MEF)			Biospheric values (BV)			Egoistic values (EV)			Perceived environmental friendliness (PEF)			Positive emotions (PE)			Negative emotions (NE)			Acceptability of innovative product (AIP)		
		95% CI[1]			95% CI[1]			95% CI[1]			95% CI[1]			95% CI[1]			95% CI[1]			95% CI[1]	
	r	LL	UL	*r*	LL	UL	*r*	LL	UL	*r*	LL	UL	*r*	LL	UL	*r*	LL	UL	*r*	LL	UL
MEF				.04	-.25	.32	-.01	-.29	.31	.91	.77	.98	.64	.38	.84	-.55	-.73	-.35	.70	.50	.85
BV	.13	-.13	.37				.33	.01	.61	.05	-.25	.35	.26	-.01	.51	.16	-.16	.41	.21	-.06	.47
EV	-.15	-.39	.12	-.11	-.38	.20				-.12	-.38	.18	.03	-.22	.31	.40	.13	.64	.00	-.24	.27
PEF	.93	.88	.97	.19	-.08	.43	-.16	-.41	.10				.75	.59	.88	-.64	-.78	-.47	.80	.68	.90
PE	.86	.76	.94	.18	-.09	.43	-.06	-.32	.18	.84	.70	.94				-.49	-.65	-.31	.86	.77	.92
NE	-.68	-.80	-.55	.02	-.22	.25	-.02	-.25	.22	-.73	-.81	-.63	-.61	-.72	-.48				-.62	-.75	-.48
AIP	.91	.84	.96	.16	-.12	.41	-.05	-.30	.19	.90	.82	.95	.87	.78	.93	-.73	-.83	-.63			

[1] BCA bootstrap confidence intervals based on 5,000 resamples.

Table SM1.2.D. Correlations between manipulated inexpensiveness, values, perceived inexpensiveness, positive and negative emotions, and acceptability in experiment III (above diagonal) and experiment IV (below diagonal)

	Manipulated inexpensiveness (MIE)			Egoistic values (EV)			Biospheric values (BV)			Perceived inexpensiveness (PIE)			Positive emotions (PE)			Negative emotions (NE)			Acceptability of innovative product (AIP)		
		95% CI[1]			95% CI[1]			95% CI[1]			95% CI[1]			95% CI[1]			95% CI[1]			95% CI[1]	
	r	LL	UL	*r*	LL	UL	*r*	LL	UL	*r*	LL	UL	*r*	LL	UL	*r*	LL	UL	*r*	LL	UL
MIE				.23	-.05	.47	-.12	-.40	.17	.87	.77	.94	.16	-.11	.45	-.22	-.40	.03	.10	-.19	.38
EV	.02	-.23	.24				-.03	-.35	.31	.17	-.12	.46	.08	-.19	.31	.10	-.28	.39	-.06	-.39	.27
BV	-.21	-.43	.03	.26	-.03	.51				.03	-.27	.31	.47	.18	.69	-.12	-.32	.13	.30	-.04	.62
PIE	.73	.57	.86	-.03	-.27	.19	.03	-.21	.25				.26	.01	.51	-.25	-.44	-.01	.13	-.14	.38
PE	.33	.09	.55	.09	-.14	.30	.16	-.10	.40	.52	.30	.70				-.24	-.48	.14	.58	.36	.74
NE	-.25	-.45	-.04	.33	.11	.52	.22	.01	.42	-.17	-.39	.01	-.02	-.23	.18				-.51	-.67	-.26
AIP	.35	.12	.55	.03	-.18	.22	.09	-.19	.36	.49	.31	.64	.70	.51	.83	-.30	-.52	-.07			

[1] BCA bootstrap confidence intervals based on 5,000 resamples.

1.3 Selection of Emotions

We selected the emotions applied in study 1 in a pilot study. First, we compiled the emotions considered in key emotion theories (e.g., Ekman, 1992; Russell, 1980) and in previous research in the field (e.g., Böhm, 2003; Huijts et al., 2007), which resulted in 53 emotions (including synonyms, such as afraid and fearful). Next, two of the authors assessed each emotion regarding their relevance for acceptability of sustainable innovations, that is, whether or not people might feel the respective emotion toward sustainable innovations ($\kappa = .60$, which indicates a moderate inter-rater agreement). In case of disagreement, we discussed the respective emotion among the study team and decided jointly on the emotion's relevance. Of the 53 emotions, 28 were assessed as potentially relevant for acceptability of sustainable innovations (still including synonyms). We applied the 28 emotions in a study among a student sample. We provided participants with short descriptions of the innovative products used in study 1 and measured participants' emotions toward and acceptability of the products. We did the same for an additional innovation, namely carbon capture and storage. We assessed the emotions based on their variance and their correlation with acceptability and removed emotions that had lower variance and/or lower correlations. Therein, we focused particularly on removing synonyms and ensured to keep a balanced number of positive and negative emotions. The remaining emotions contained six positive emotions, namely comfortable, excited, happy, optimistic, relieved, and satisfied, and seven negative emotions, namely afraid, angry, disappointed, disgusted, powerless, upset, and worried.

1.4 Acceptability Measure

Table SM1.4. Semantic differential scales measuring acceptability of innovations.

	Poles and mid-points of the 9-point semantic differential scales[1]		
Item number	1	5	9
1	Very negative	Neither negative nor positive	Very positive
2	Very senseless	Neither senseless nor sensible	Very sensible
3	Very undesirable	Neither undesirable nor desirable	Very desirable
4	Very unattractive	Neither unattractive nor attractive	Very attractive
5	Very useless	Neither useless nor useful	Very useful
6	Very unnecessary	Neither unnecessary nor necessary	Very necessary
7	Very unacceptable	Neither unacceptable nor acceptable	Very acceptable
8	Very intolerable	Neither intolerable nor tolerable	Very tolerable

[1] Scales were introduced with the sentence "According to me, [microalgae-based foods/nanophotonic lightbulbs] are…"

1.5 Effects of Manipulated Innovation-Characteristics on Corresponding Perceived Innovation-Characteristics and the Role of Values

Table SM1.5.A. Regression coefficients of innovations' manipulated environmental friendliness, values, and their interactions on innovations' perceived environmental friendliness.

	Experiment I (n = 50): Microalgae-based foods									Experiment II (n = 52): Nanophotonic lightbulbs								
IV[3]	*B*	95% CI[1,2]		*SE*	β	ΔR^2	ΔF	R^2	*F*	*B*	95% CI[1,2]		*SE*	β	ΔR^2	ΔF	R^2	*F*
Step 2[4]						.82	78.63***	.84	60.56***						.87	113.26***	.88	85.47***
Constant	1.89	1.37	2.61	0.36						1.55	1.25	1.88	0.17					
OE[5]	-0.37	-0.82	-0.01	0.21	-.11					-0.07	-0.37	0.28	0.16	-.02				
MEF[6]	5.93	5.18	6.52	0.41	.91					6.03	5.46	6.57	0.32	.93				
BV[7]	0.14	-0.20	0.79	0.29	.04					0.08	-0.27	0.46	0.19	.02				
MEF[6]*BV[7]	-0.23	-1.06	0.42	0.36	-.05					0.21	-0.39	0.84	0.30	.05				
Step 3						.01	1.99	.86	42.81***						.00	0.20	.88	55.11***
EV[8]	-0.48	-1.57	0.18	0.44	-.14					-0.01	-0.36	0.19	0.17	.00				
MEF[6]*EV[8]	0.15	-0.75	1.35	0.54	.03					-0.16	-1.10	0.72	0.42	-.03				

[1] BCA bootstrap confidence intervals based on 5,000 resamples. [2] 90% CIs are presented for the effect of manipulated on perceived environmental friendliness. [3] Independent variable. [4] Estimates of steps 1, in which we controlled for the influence of order of experiments on perception and for which the *F*-test of the overall significance of the model were non-significant, are not displayed. [5] Order of experiment. [6] Manipulated environmental friendliness (0 = environmentally unfriendly, 1 = environmentally friendly). [7] Biospheric values. [8] Egoistic values. *** $p \leq .001$.

Table 1.5.B. Regression coefficients of innovations' manipulated inexpensiveness, values, and their interaction on innovations' perceived inexpensiveness.

	Experiment III (n = 48): Microalgae-based foods									Experiment IV (n = 44): Nanophotonic lightbulbs								
IV[3]	*B*	95% CI[1, 2]		*SE*	β	ΔR^2	ΔF	R^2	*F*	*B*	95% CI[1, 2]		*SE*	β	ΔR^2	ΔF	R^2	*F*
Step 2[4]						.76	45.47***	.76	34.14***						.68	28.71***	.69	21.79***
Constant	1.87	1.51	2.30	0.19						2.27	1.80	2.71	0.24					
OE[5]	0.00	-0.40	0.32	0.21	.00					-0.18	-0.60	0.28	0.22	-.07				
MIE[6]	4.76	3.97	5.44	0.46	.87					4.41	3.58	5.23	0.44	.83				
EV[7]	-0.27	-0.62	0.15	0.19	-.10					0.03	-0.44	0.52	0.24	.01				
MIE[6]*EV[7]	0.43	-0.43	2.26	0.56	.11					0.35	-0.60	1.22	0.48	.11				
Step 3						.03	2.72	.79	25.49***						.04	2.43	.73	16.40***
BV[8]	0.30	-0.09	0.63	0.18	0.11					0.12	-0.54	1.10	0.32	.04				
MIE[6]*BV[8]	0.41	-0.59	1.32	0.52	0.09					0.84	-0.28	1.86	0.63	.23				

[1] BCA bootstrap confidence intervals based on 5,000 resamples. [2] 90% CIs are presented for the effect of manipulated on perceived inexpensiveness. [3] Independent variable. [4] Estimates of steps 1, in which we controlled for the influence of order of experiments on perception and for which the *F*-test of the overall significance of the model were non-significant, are not displayed. [5] Order of experiment. [6] Manipulated inexpensiveness (0 = expensive, 1 = inexpensive). [7] Egoistic values. [8] Biospheric values. *** $p \leq .001$.

1.6 Testing the VICE Model for Manipulated Innovation-Characteristics

Table SM1.6.A. Regression coefficients of manipulated environmental friendliness, values, and their interactions on positive and negative emotions toward the innovations.

	Experiment I (*n* = 50): Microalgae-based foods										Experiment II (*n* = 52): Nanophotonic lightbulbs									
	Positive emotions					Negative emotions					Positive emotions					Negative emotions				
		95% CI[1, 2]					95% CI[1, 2]					95% CI[1, 2]					95% CI[1, 2]			
IV[3]	*B*	LL	UL	*SE*	β	*B*	LL	UL	*SE*	β	*B*	LL	UL	*SE*	β	*B*	LL	UL	*SE*	β
Step 2[4]																				
Constant	1.85	1.45	2.33	0.25		2.81	2.37	3.21	0.22		1.20	1.07	1.36	0.08		2.61	2.21	3.02	0.21	
Order of exp.	0.06	-0.30	0.39	0.16	.04	0.03	-0.26	0.34	0.13	.03	-0.11	-0.37	0.14	0.13	-.06	0.01	-0.19	0.20	0.10	.01
MEF[5]	1.77	1.24	2.26	0.31	.63	-1.20	-1.64	-0.75	0.26	-.55	2.90	2.48	3.30	0.23	.85	-1.40	-1.76	-1.05	0.23	-.70
BV[6]	0.21	-0.13	0.69	0.22	.15	0.46	0.07	0.88	0.21	.42	-0.14	-0.35	0.04	0.10	-.08	0.38	0.03	0.78	0.19	.38
MEF[5]*BV[6]	0.28	-0.17	0.69	0.27	.13	-0.62	-1.01	-0.30	0.24	-.37	0.53	0.18	0.88	0.22	.23	-0.50	-0.85	-0.20	0.20	-.36
Model fit	ΔR^2 = .47, $\Delta F(3,45)$ = 13.44***<br>R^2 = .47, $F(4,45)$ = 10.09***					ΔR^2 = .41, $\Delta F(3,45)$ = 10.46***<br>R^2 = .41, $F(4,45)$ = 7.89***					ΔR^2 = .75, $\Delta F(3,47)$ = 52.50***<br>R^2 = .78, $F(4,47)$ = 40.60***					ΔR^2 = 52., $\Delta F(3,47)$ = 17.54***<br>R^2 = .54, $F(4,47)$ = 13.58***				
Step 3																				
EV[7]	-0.11	-0.80	0.57	0.30	-.08	0.65	0.26	0.98	0.19	.59	0.13	-0.03	0.26	0.08	.08	-0.07	-0.35	0.42	0.17	-.07
MEF[5]*EV[7]	0.09	-0.58	0.74	0.39	.04	-0.44	-0.92	0.14	0.26	-.29	-0.15	-0.86	0.48	0.31	-.06	0.01	-0.39	0.28	0.19	.01
Model fit	ΔR^2 = .00, $\Delta F(2,43)$ = 0.11<br>R^2 = .48, $F(6,43)$ = 6.50***					ΔR^2 = .16, $\Delta F(2,43)$ = 8.21**<br>R^2 = .57, $F(6,43)$ = 9.68***					ΔR^2 = .00, $\Delta F(2,45)$ = 0.32<br>R^2 = .78, $F(6,45)$ = 26.40***					ΔR^2 = .00, $\Delta F(2,45)$ = 0.21<br>R^2 = .54, $F(6,45)$ = 8.81***				

[1] BCA bootstrap confidence intervals based on 5,000 resamples. [2] 90% CIs are presented for manipulated environmental friendliness and its interaction with biospheric values. [3] Independent variable. [4] Estimates of steps 1, in which we controlled for the influence of order of experiments on positive and negative emotions and for which the *F*-test of the overall significance of the model were non-significant, are not displayed here. [5] Manipulated environmental friendliness. [6] Biospheric values. [7] Egoistic values. ** $p \leq .01$, *** $p \leq .001$.

Table SM1.6.B. Regression coefficients of manipulated inexpensiveness, values, and their interactions on positive and negative emotions toward the innovations.

	Experiment III (*n* = 44): Microalgae-based foods										Experiment IV (*n* = 48): Nanophotonic lightbulbs									
	Positive emotions					Negative emotions					Positive emotions					Negative emotions				
		95% CI[1, 2]					95% CI[1, 2]					95% CI[1, 2]					95% CI[1, 2]			
IV[3]	*B*	LL	UL	*SE*	β	*B*	LL	UL	*SE*	β	*B*	LL	UL	*SE*	β	*B*	LL	UL	*SE*	β
Step 2[4]																				
Constant	2.70	2.20	3.28	0.27		1.65	1.36	1.96	0.16		2.74	2.22	3.18	0.27		1.65	1.39	1.92	0.14	
Order of exp.	0.00	-0.38	0.42	0.19	.00	0.13	-0.02	0.29	0.09	.23	0.03	-0.36	0.44	0.19	.02	0.00	-0.15	0.14	0.07	-.01
MIE[5]	0.38	-0.27	1.05	0.40	.15	-0.29	-0.63	0.05	0.18	-.24	1.14	0.48	1.83	0.37	.43	-0.46	-0.73	-0.20	0.16	-.46
EV[6]	0.00	-0.61	0.56	0.31	.00	0.18	-0.24	0.60	0.22	.30	-0.17	-0.81	0.43	0.33	-.13	0.07	-0.22	0.45	0.15	.14
MIE[5]*EV[6]	0.10	-0.54	0.78	0.40	.06	-0.16	-0.59	0.26	0.24	-.18	0.53	-0.03	1.04	0.39	.32	0.01	-0.25	0.24	0.17	.02
Model fit	ΔR^2 = .03, $\Delta F(3,43)$ = 0.44<br>R^2 = .03, $F(4,43)$ = 0.33					ΔR^2 = .09, $\Delta F(3,43)$ = 1.45<br>R^2 = .14, $F(4,43)$ = 1.70					ΔR^2 = .24, $\Delta F(3,39)$ = 4.02*<br>R^2 = .24, $F(4,39)$ = 3.02*					ΔR^2 = .25, $\Delta F(3,39)$ = 4.40**<br>R^2 = .25, $F(4,39)$ = 3.31*				
Step 3																				
BV[7]	0.63	0.07	1.06	0.27	.51	-0.22	-0.55	0.10	0.15	-.37	0.19	-0.67	1.10	0.41	.15	0.13	-0.29	0.48	0.18	.25
MIE[5]*BV[7]	0.24	-0.53	1.19	0.39	.12	0.18	-0.11	0.48	0.16	.19	0.10	-0.97	0.98	0.60	.06	-0.16	-0.58	0.28	0.23	-.23
Model fit	ΔR^2 =.29, $\Delta F(2,41)$ = 8.96**<br>R^2 = .32, $F(6,41)$ = 3.29*					ΔR^2 = .08, $\Delta F(2,41)$ = 2.09<br>R^2 = .22, $F(6,41)$ = 1.89 ^{t}					ΔR^2 = .02, $\Delta F(2,37)$ = 0.57<br>R^2 = .26, $F(6,37)$ = 2.16 ^{t}					ΔR^2 = .03, $\Delta F(2,37)$ = 0.66<br>R^2 = .28, $F(6,37)$ = 2.38*				

[1] BCA bootstrap confidence intervals based on 5,000 resamples. [2] 90% CIs are presented for manipulated inexpensiveness and its interaction with egoistic values. [3] Independent variable. [4] Estimates of steps 1, in which we controlled for the influence of order of experiments on positive and negative emotions and for which the *F*-test of the overall significance of the model was non-significant, are not displayed here. [5] Manipulated inexpensiveness. [6] Egoistic values. [7] Biospheric values. ^{t} = $p \le .10$ * $p \le .05$, ** $p \le .01$, *** $p \le .001$.

2 Supplementary Material of Study 2

2.1 Information Texts

Table SM2.1. Information provided on algae-based biofuels

Section/Factor	Conditions	Texts
General introduction	All conditions	Renewable energy is retrieved from renewable resources, which are naturally replenished. **Algae** is a renewable energy source, from which biofuel can be made. **Algae-based biofuel** is an increasingly popular form of renewable energy. It is arguably a solution to the world's growing energy demand because algae is one of the fastest growing plants.
Factor 1	Condition 1: Considered	Governments across Europe are currently **considering implementing** this energy source.
	Condition 2: Implemented	Governments across Europe **already started to implement** this energy source.
General information	All conditions	Algae-based biofuel emits less CO_2 than fossil fuels, such as coal and oil.
Factor 2	Condition 1: Environmentally unfriendly	However, it still **emits a substantial amount of CO_2** by the burning process that is required for energy generation. Additionally, in order to produce enough energy, algae requires huge areas of land and thus substantially contributes to the **clearing of forests** and **destruction of natural areas**. Another negative side effect is that algae **pollutes** water and land through poisonous fertilizers and pesticides that are used to grow it.
	Condition 2: Environmentally friendly	Also, like any other plant it **reduces CO_2** from the atmosphere when grown in sunlight and releases oxygen, thereby depolluting the air. Additionally, algae can be grown in any type of water, including sweet water, natural water or wastewater, and therefore **does not require clearing of forests and destruction of natural areas**. Another positive side effect is that algae **depollutes the water** through absorbing and removing contaminants.

Emphases are presented as in the original text.

2.2 Descriptive Statistics and Intercorrelations of Variables Tested in Study 2

Table SM2.2.A. Means and standard deviations of perceived environmental friendliness, positive and negative emotions and acceptability per experimental condition.

	Condition[1]	*n*	*M*	*SD*	*t*
Biospheric values	1	121	4.71	1.57	$t(244) = 0.21$
	2	125	4.67	1.56	
Egoistic values	1	121	3.01	1.38	$t(244) = 1.38$
	2	125	2.78	1.22	
Perceived environmental friendliness	1	121	3.53	1.26	$t(234.20) = -17.61$***
	2	125	6.14	1.06	
Positive emotions	1	121	2.04	0.85	$t(244) = -11.15$***
	2	125	3.26	0.86	
Negative emotions	1	121	2.17	0.92	$t(208.11) = 8.19$***
	2	125	1.35	0.62	
Acceptability	1	121	4.03	1.08	$t(244) = -15.37$***
	2	125	5.98	0.90	

[1] Condition 1: Environmentally unfriendly. Condition 2: Environmentally friendly. ** $p \leq .01$. *** $p \leq .001$.

Table SM2.2.B. Correlations between manipulated environmental friendliness, values, perceived environmental friendliness, positive and negative emotions, and acceptability of algae-based biofuel

	Manipulated environmental friendliness			Biospheric values			Egoistic values			Perceived environmental friendliness			Positive emotions			Negative emotions		
		95% CI[1]			95% CI[1]			95% CI[1]			95% CI[1]			95% CI[1]			95% CI[1]	
	r	LL	UL	*r*	LL	UL	*r*	LL	UL	*r*	LL	UL	*r*	LL	UL	*r*	LL	UL
Biospheric values	-.01	-.14	.11															
Egoistic values	-.09	-.21	.04	-.06	-.19	.07												
PEF[2]	.75	.68	.81	-.05	-.17	.08	-.12	-.24	.00									
Positive emotions	.58	.50	.66	.13	.01	.26	-.01	-.13	.10	.63	.54	.72						
Negative emotions	-.46	-.56	-.36	.16	.04	.27	.08	-.05	.21	-.57	-.66	-.46	-.34	-.45	-.24			
Acceptability	.70	.64	.76	.07	-.06	.19	-.09	-.21	.02	.79	.72	.86	.75	.68	.81	-.64	-.72	-.55

[1] BCA bootstrap confidence intervals based on 5,000 resamples. [2] Perceived environmental friendliness.

2.3 Effects of Manipulated Environmental Friendliness of Algae-Based Biofuel on Perceived Environmental friendliness and the Role of Values

Table SM2.3. Regression coefficients of manipulated environmental friendliness of algae-based biofuel, values, and their interactions on perceived environmental friendliness of algae-based biofuel.

		95% CI of B[1, 2]							
Independent variable	B	LL	UL	SE	β	ΔR^2	ΔF	R^2	F
Step 2[3]						.56	104.60***	.57	78.67***
Constant	3.79	3.29	4.29	0.26					
Type of recruitment	-0.21	-0.58	0.16	0.19	-.05				
MEF[4]	2.61	2.35	2.87	0.14	.75				
BV[5]	-0.16	-0.39	0.06	0.11	-.09				
MEF[4]*BV[5]	0.19	-0.07	0.46	0.14	.08				
Step 3						.00	0.85	.57	52.67***
EV[6]	-0.04	-0.24	0.18	0.11	-.02				
MEF[4]*EV[6]	-0.10	-0.38	0.18	0.14	-.04				

N = 246. [1] BCA bootstrap confidence intervals based on 5,000 resamples. [2] 90% CIs are presented for the effect of manipulated on perceived environmental friendliness. [3] Estimates of step 1, which controlled for the influence of type of recruitment and reward on acceptability, and for which the F-test of the overall significance of the model were non-significant, are not displayed here. [4] Manipulated environmental friendliness (0 = environmentally unfriendly, 1 = environmentally friendly). [5] Biospheric values. [6] Egoistic values. *** $p \leq .001$.

2.4 Testing the VICE Model for Manipulated Innovation-Characteristics

Table SM2.4. Regression coefficients of manipulated environmental friendliness of algae-based biofuel, values, and their interactions on positive and negative emotions toward algae-based biofuel

	Positive emotions					Negative emotions				
		95% CI[1, 2]					95% CI[1, 2]			
Independent variable	*B*	LL	UL	*SE*	β	*B*	LL	UL	*SE*	β
Step 2[3]										
Constant	2.01	1.67	2.35	0.18		2.26	1.95	2.57	0.16	
Type of recruitment	0.03	-0.25	0.31	0.14	.01	-0.07	-0.30	0.17	0.11	-0.03
MEF[4]	1.22	1.05	1.40	0.11	.58	-0.81	-0.98	-0.65	0.10	-0.46
BV[5]	0.06	-0.10	0.21	0.08	.06	0.23	0.07	0.39	0.08	0.26
MEF[4]*BV[5]	0.17	-0.02	0.35	0.11	.12	-0.20	-0.37	-0.03	0.10	-0.16
Model fit	$\Delta R^2 = .36$, $\Delta F(3,241) = 46.00$*** $R^2 = .36$, $F(4,241) = 34.56$***					$\Delta R^2 = .25$, $\Delta F(3,241) = 26.99$*** $R^2 = .25$, $F(4,241) = 20.38$***				
Step 3										
EV[6]	0.05	-0.09	0.19	0.07	.05	-0.08	-0.23	0.07	0.08	-0.08
MEF[4]*EV[6]	0.01	-0.19	0.21	0.10	.01	0.26	0.06	0.49	0.10	0.19
Model fit	$\Delta R^2 = .00.$, $\Delta F(2,239) = 0.52$ $R^2 = .37$, $F(6,239) = 23.12$***					$\Delta R^2 = .02.$, $\Delta F(2,239) = 3.69$* $R^2 = .27$, $F(6,239) = 15.12$***				

[1] BCA bootstrap confidence intervals based on 5,000 resamples. [2] 90% CIs are presented for manipulated environmental friendliness and its interaction with biospheric values. [3] Estimates of steps 1, which controlled for the influence of type of recruitment and reward on acceptability, and for which the *F*-test of the overall significance of the model were non-significant, are not displayed here. [4] Manipulated environmental friendliness. [5] Biospheric values. [6] Egoistic values. *** $p \leq .001$.

3 References

Böhm, G. (2003). Emotional reactions to environmental risks: Consequentialist versus ethical evaluation. *Journal of environmental psychology, 23*(2), 199-212. doi:10.1016/S0272-4944(02)00114-7

Ekman, P. (1992). Are there basic emotions? *Psychological Review, 99*(3), 550-553. doi:10.1037/0033-295X.99.3.550

Huijts, N. M. A., Midden, C. J. H., & Meijnders, A. L. (2007). Social acceptance of carbon dioxide storage. *Energy Policy, 35*(5), 2780-2789. doi:10.1016/j.enpol.2006.12.007

Russell, J. A. (1980). A circumplex model of affect. *Journal of Personality and Social Psychology, 39*(6), 1161-1178. doi:10.1037/h0077714
